# Mitochondria-specific photorelease of ceramide induces apoptosis

**DOI:** 10.1016/j.jlr.2025.100907

**Published:** 2025-09-19

**Authors:** Christian Schröer, Matthijs Kol, Anna Koch, Emely Döffinger, Murali Annamalai, Joost C.M. Holthuis

**Affiliations:** 1 Division of Molecular Cell Biology, Department of Biology/Chemistry, Osnabrück University, Osnabrück, Germany; 2 Center for Cellular Nanoanalytics, Osnabrück University, Osnabrück, Germany

**Keywords:** caspase-9, chemical synthesis, click chemistry, inner mitochondrial membrane, mitochondrial apoptosis, photocage, sphingolipids

## Abstract

Deciphering the mechanisms by which bioactive intermediates of lipid metabolism influence cell behavior is a challenging task. We previously demonstrated that de novo synthesized ceramides are authentic transducers of apoptosis and that their CERT-mediated diversion to mitochondria is sufficient to initiate BAX-dependent apoptosis. To further unravel the mechanism by which mitochondrial ceramides commit cells to death, we here developed a novel mitochondria-targeted and photocaged short-chain ceramide with a clickable alkyne group for derivatization with a fluorescent reporter. We show that this compound readily and selectively accumulates inside mitochondria in a biologically inert state. Subsequent photorelease of the ceramide moiety triggered apoptosis as evidenced by proteolytic cleavage of central components of the caspase-dependent cell death pathway. Our findings reinforce the notion that ceramides can initiate apoptotic cell death by acting directly on mitochondria and establish mitochondria-targeted photocaged ceramides as novel tools to elucidate the underlying mechanism with the spatiotemporal precision of light.

Apoptosis is the best characterized form of regulated cell death with a central role in organismal development and tissue homeostasis. Dysregulation of apoptosis contributes to disease, notably cancer and autoimmunity. Apoptosis triggered by stress stimuli (e.g. DNA damage, cytokines, and cytostatics) involves different pathways that converge on mitochondria, culminating in permeabilization of the outer mitochondrial membrane (OMM) and cytosolic release of cytochrome *c* and other apoptotic intermembrane space proteins (1). In the cytosol, cytochrome *c* binds to apoptotic protease factor-1 (APAF1) and recruits pro-caspase-9 to form the apoptosome, resulting in proteolytic activation of caspase-9 (Casp9). Activated Casp9 then proteolytically activates Casp3 and Casp7, which ultimately execute an ordered self-destruction of the cell (2, 3). Permeabilization of the OMM is tightly controlled by pro- and anti-apoptotic members of the B-cell lymphoma 2 (BCL2) family of proteins (4). In particular, the anti-apoptotic BCL2 proteins counter the effects of the pro-apoptotic BCL2 proteins BAX and BAK, which form cytochrome *c*-conducting pores in the OMM to initiate caspase activation and cell death (5, 6). While research into the activation mechanisms of BAX and BAK initially focused on pro-apoptotic BH3-only BCL2 proteins, there is also evidence that alterations in the OMM lipid composition play a direct and critical role (7, 8).

Ceramides are central intermediates of sphingolipid metabolism that serve as precursors for the production of sphingomyelin (SM) and glycosphingolipids (9). Numerous studies have implicated ceramides as potent mediators of stress-induced mitochondrial apoptosis (10). Cellular ceramide levels rise concomitantly with apoptosis induction in response to different stress stimuli, including chemotherapeutic agents, death receptor activation or irradiation, either via an enhanced de novo ceramide biosynthesis in the ER or through activation of sphingomyelinases (SMases) (11, 12, 13, 14). Suppression of ceramide accumulation renders cells resistant to these apoptotic stimuli, in line with a direct role of ceramides in stress-induced apoptosis. However, the exact mechanism by which ceramides exert their apoptogenic activities remains a subject of debate. Some studies suggest that ceramides act directly on the OMM, where they form stable channels that facilitate the release of cytochrome *c* (15, 16). Another model postulates that ceramides generated in the OMM upon radiation of mammalian cells form microdomains into which BAX inserts and oligomerizes into a cytochrome *c*-conducting pore (17). In addition, experiments with purified mitochondria indicate that ceramides may require metabolic conversion to gain apoptogenic activity (18). Addressing the actual contribution of mitochondrial ceramides to the apoptotic response in living cells is challenging, as ceramides are readily metabolized into various other bioactive lipid species, which can influence stress resistance through multiple pathways (19).

Ceramides are synthesized de novo by *N*-acylation of sphingoid long-chain bases on the cytosolic surface of the ER. In mammalian cells, the bulk of newly synthesized ceramides is delivered to the *trans-*Golgi by ceramide transfer protein CERT (20) for metabolic conversion into SM by *trans*-Golgi-resident SM synthases (21). To study the consequences of targeting the biosynthetic ceramide flow to mitochondria, we previously created a CERT variant equipped with an OMM anchor, *mito*CERT. To achieve increased temporal control over mitochondrial ceramide transport, we also developed a rapamycin-inducible variant of mitoCERT, designated as *s*CERT. We showed that *mito*CERT- and *s*CERT-mediated delivery of newly synthesized ceramides to mitochondria triggers BAX-dependent apoptosis (22, 23). Apoptosis induction was blocked by targeting a bacterial ceramidase to mitochondria, indicating that apoptogenic activity strictly relied on intact ceramides rather than metabolic intermediates of ceramide turnover. A subsequent chemical screen for ceramide-binding proteins combined with molecular dynamics simulations and functional studies in cancer cells yielded the voltage-dependent anion channel VDAC2 as a critical effector of ceramide-mediated apoptosis (24). Other work revealed that VDAC2 specifies BAX recruitment to mitochondria and controls the apoptogenic activity of BAX by mediating its retro-translocation into the cytosol (25). We recently observed that VDAC residues involved in ceramide binding also directly participate in mobilizing hexokinase-I (HKI) to mitochondria (26), a condition that promotes cell growth and survival in hyperglycolytic tumors (27). Together, these results point to a potential mechanism by which mitochondrial ceramides exert their anti-neoplastic activity.

So far, most approaches for studying ceramide-activated cell death pathways rely on external addition of truncated ceramide analogs, treatment of cells with apoptotic stimuli that affect ceramide pools in multiple organelles or redirecting intracellular ceramide trafficking with modified lipid transfer proteins. A major drawback of these methods is that they lack precision or require extensive protein engineering. Recently, photocaged lipids have emerged as alternative experimental tools that offer a non-invasive way to manipulate lipid pools on a subcellular scale and with high temporal precision (28, 29, 30). These compounds carry a photo-labile protecting group that blocks their biological activity until the active lipid is released upon a flash of light. Additional precision is achieved by modifying the photocage with organelle-targeting moieties. The first example of such organelle-targeted caged lipid probes were arachidonic acid derivatives directed to the PM (31). Subsequent studies extended this approach to other organelles and lipids (32), including lysosome- (33, 34) and mitochondria-targeted sphingosine (35).

Here, we report on a novel mitochondrially-targeted and photocaged short-chain ceramide with a clickable alkyne group for derivatization with an azide-containing fluorophore. We demonstrate that this compound readily and selectively accumulates in mitochondria and executes its apoptogenic activity upon a short flash of UV-light while staying inactive in the dark. With this, we established a novel tool to further unravel the tumor suppressor activities of ceramides at a mechanistic level.

## Materials and Methods

### Chemical synthesis of cgMito-cCer_6_

The complete chemical synthesis of cgMito-cCer_6_ is described in the Supplementary Information.

## Reagents and Antibodies

All chemicals were purchased from AppliChem, BioRad, Carl Roth, Enzo Life Sciences, Merck, Sigma Aldrich, or ThermoFisher Scientific. AlexaFluor™ 647-azide was from ThermoFisher Scientific (cat. no. A10277). Staurosporine was from Cayman Chemical (cat. no. 81590). Dulbecco’s Modified Eagle’s medium (DMEM, +4.5 g/l glucose), fetal calf serum (FCS) and Trypsin/EDTA were from PAN Biotech, Opti-MEM was from Gibco. Penicillin/Streptomycin (Pen/Str 10.000 U/ml) was from PAN Biotech (cat. no. P06-07100). Linear polyethylenimine (PEI) MAX 40k was from Polysciences Inc. (cat. no. 24765-1). Antibodies used were mouse monoclonal anti-PARP1 (Santa Cruz Biotechnology, #Sc-8007; IB 1:1000), mouse monoclonal anti-Caspase-9 (Cell Signaling Technology, #9508; IB 1:000) and HRP-conjugated goat anti-mouse IgG (Thermo Fisher Scientific, #31430; IB 1:4000 in PBST.

### DNA constructs

Expression constructs pSEMS-TOM20-eGFP and pSEMS-VAPA-mCherry are described in (22). pSEMS-CIR-dsRed was a kind gift from Karin Busch (University of Münster) and is described in (36).

### Cell culture and transfection

Human cervical carcinoma (HeLa) cells (ATCC CCL-2) were cultured in Dulbecco’s modified Eagle’s medium (DMEM), which was supplemented with 4.5 g/L glucose, 2 mM L-glutamine and 10% fetal bovine serum (FBS). Cells were grown in 10 cm dishes and passaged at a maximum confluency of 80% to prevent overgrowth. For transfection, plasmid DNA was mixed with PEI MAX 40K (1 mg/ml) in 150 mM NaCl. For 8-well microscopy slides (IBIDI), per well 0.45 μg plasmid DNA was diluted in 30 μl NaCl and mixed with 0.9 μl PEI. The transfection mix was vortexed and after 15 min incubation at RT added dropwise to the cells. Cells were incubated at 37°C for 4–5 h with transfection mix, washed twice with PBS and then incubated in fresh culture medium supplemented with 100 U/ml of Pen/Strep overnight.

### Absorbance measurements

For absorbance measurements, 16 μM of cgMito-cCer_6_ in 240 μl MeOH was illuminated for the indicated times with blue-light (470 nm) or UV-A-light (365 nm) in a UV-cuvette (Brand) using a CoolLED pE-300 setup (CoolLED, UK) at 80% power output. After illumination, UV-Vis absorbance was measured using a BioSpectrophotometer (Eppendorf). All steps were performed in the dark to minimize exposure of the photocaged compound to visible light.

### Uncaging assay

To evaluate the photo-cleavage efficiency of the mitochondria-targeted caged ceramide, 50 μM of cgMito-cCer_6_ in 120 μl MeOH was subjected to Blue-light (470 nm) or UV-A-light (365 nm) for the indicated times in brown glass vials using a CoolLED pE-300 setup (CoolLED) at 80% power output. After each time point, a 30 μl sample was transferred to a fresh Eppendorf Safelock tube (cat. no. 0030121023) and dried down in a Christ RVC 2–18 speedvac (Christ) equipped with a Vacuubrand MZ 2C diaphragm vacuum pump (Vacuubrand) for 40 min at 40°C. The alkyne group was derivatized with AlexaFluor647-azide by Cu(I)-catalyzed click reaction as previously described (37). To this end, the lipid film was resuspended in 7 μl CHCl_3_. For click reactions, the azide-alkyne ratio was kept 1:1. A master click-mix was prepared (based on 10 reactions, each containing 6 nmol alkyne), containing 60 nmol AlexaFluor647-azide in 68 μl 10 mM tetrakis- (acetonitrile)copper(I) tetrafluoroborate (Sigma, cat. no. 677892) plus 230 μl pure EtOH. To each sample, 30 μl of click-mix was added. After vortexing, the samples were briefly spun down and incubated in the dark for 4 h at 43°C in a heat block without shaking to ensure complete condensation of the organic solvent in the lids of the tubes. Reaction mixtures were briefly centrifuged (21.000 *g*, 30 s), diluted with 30 μl CHCl_3_, and transferred to brown HPTLC vials with microinserts and sealed with aluminum crimp closure lids (Macherey & Nagel) before TLC analysis.

### Uptake assay

HeLa cells were seeded at a density of 500.000 into 6-well dishes and grown overnight. The next day, the cells were washed once with PBS and incubated in 500 μl Opti-MEM containing 10 μM cgMito-cCer_6_ or cgMito, corresponding to 5 nmol of total compound, which was added directly to the cells from an ethanolic stock (2 mM). Cells incubated in the absence of cgMito-cCer_6_ or cgMito served as reference samples. After 0, 1, 4 or 24 h of incubation, the medium was collected and transferred to brown glass vials containing 1.9 ml CHCl_3_:MeOH (1:2, v:v) and vortexed. The cells were harvested by trypsinization and resuspended in 1 ml PBS supplemented with protease inhibitor cocktail (PIC; 1 μg/ml aproptonin, 1 μg/ml leupeptin, 1 μg/ml pepstatin, 5 μg/ml antipain and 157 μg/ml benzamidine) followed by centrifugation at 900 *g* for 10 min at at 4°C. After removal of 900 μl of the supernatant, the cell pellet was resuspended in the remaining 100 μl and 375 μl CHCl_3_:MeOH (1:2, v:v) was added to precipitate protein and halt any further metabolic reactions. To the input reference samples, 5 nmol of cgMito-cCer_6_ or cgMito was added prior to lipid extraction to correct for extraction efficiency. All samples were stored at −20°C overnight prior to lipid extraction.

### Lipid extraction

Lipid extractions were performed in Eppendorf Protein LoBind tubes according to the Bligh and Dyer method (38). Extracts were centrifuged at 21.000 *g* for 5 min at 4°C and the supernatants were transferred to fresh tubes containing one vol. CHCl_3_ and 1.25 vol. 0.45% NaCl to induce phase separation. After vigorous vortexing for 5 min at RT and subsequent centrifugation at 21.000 *g* for 5 min at 4°C, the organic phase was transferred to a fresh tube containing 3.75 vol. MeOH:0.45% NaCl (1:1, v:v). After vigorous vortexing for 5 min at RT and subsequent centrifugation (21.000 *g*, 5 min, 4°C), the organic phase was transferred to fresh tubes and dried to a lipid film in a Christ RVC 2–18 speedvac (Christ) equipped with a Vacuubrand MZ 2C diaphragm vacuum pump (Vacuubrand) at 40°C for 40 min. The lipid film was resuspended in 100 μl CHCl_3_:MeOH (1:1, v:v) and subjected to TLC analysis.

### TLC analysis

Lipid extracts were applied on NANO-ADAMANT HP TLC plates (Machery & Nagel) using a Camag ATS4 TLC sampler (CAMAG, Germany). AlexaFluor647-labeled lipids were separated in CHCl_3_:MeOH:H_2_O:HAc (65:25:4:1, v:v:v:v) using a CAMAG ADC2 automatic TLC developer (CAMAG). AlexaFluor647-labeled lipids were visualized using a Typhoon FLA 9500 Biomolecular Imager (GE Healthcare Life Sciences) with a 647 nm excitation laser, LPB filter, 50 μm pixel size, and PMT voltage set to 290 V. Unlabeled lipids were separated in CH_2_Cl_2_:AcOEt:MeOH (40:20:15, v:v:v), and coumarin fluorescence was detected using a CoolLED pE-300 light source or ChemiDoc (BioRad) scanner with trans UV-illumination, adjusting exposure times as needed.

### Live cell imaging

For live cell imaging experiments, HeLa cells were seeded 48 h prior to the experiment in μ-Slide 8-well chambers (IBIDI) at a density of 20 x 10^4^ cells. The following day, cells were transfected with the indicated organellar markers. For co-localization experiments, cells were incubated the next day for 1 h in Opti-MEM containing cgMito-cCer_6_, which was added from an ethanolic stock (2 mM) to a final concentration of 5 μM. Before imaging, cells were washed once with PBS and fresh Opti-MEM containing 100 U/ml Pen/Strep was added to the wells. To induce hypotonic swelling of organelles, the medium was changed to 1% Opti-MEM in H_2_O followed by 5 min incubation on the microscopy stage before imaging. Live cell imaging was performed using a Zeiss Cell Observer Z1 confocal microscope (Zeiss) equipped with a CSU-X1a 5000 Spinning Disk unit (Yokogawa), a Evolve EMCDD camera (Photonics) and an Alpha Plan-Apochromat 63x/1.46 NA oil immersion objective (Zeiss) on a motorized xyz-stage PZ-2000 XYZ (Applied Scientific Instrumentation). For imaging, the following filter sets were used: Blue emission with BP 445/50 (range: 420–470 nm), green emission with BP 525/50 (range: 500–550 nm), orange emission with BP 605/70 (range: 570–640 nm) and red emission with BP 690/50 (range 665–715 nm). During live cell imaging, cells were maintained at 37°C using a TempModule S1 (Zeiss). All images were acquired using Zeiss Zen 2012 software and further processed and analyzed using Fiji software (National Institute of Health). Z-stacks of 0.2 μm were taken across 21 optical slices.

### Immunoblot analysis

Unless indicated otherwise, 500.000 HeLa cells were seeded into 6-well cell culture dishes and grown overnight. The next day, cells were washed with PBS and incubated with 500 μl Opti-MEM containing 10 μm cgMito-cCer_6_, which was added directly from an ethanolic stock (2 mM). Cells incubated with Opti-MEM containing an equal volume of EtOH served as vehicle control. After 1 h of incubation at 37°C in the dark, cells were washed with PBS to remove any non-internalized compound, and 1 ml of fresh Opti-MEM was added. Next, cells were either kept in the dark or illuminated with a CoolLED pE-300 light source at a maximum wavelength of 365 nm at 80% power output for 30 s. To this end, the lid of the 6-well plate was removed, and the light source was positioned at 25 mm distance from the bottom of the well. To ensure homogenous illumination of the cells, the 6-well plate was placed on a rotating device. After illumination, cells were further incubated in fresh Opti-Mem for 4 h at 37°C in the dark. Cells incubated with staurosporin (1 μM, 4 h) served as a control. To harvest the cells for immunoblot analysis, the culture medium was collected in 15 ml Falcon tubes on ice. Next, the cells were washed once with ice-cold PBS and the wash was combined with the collected medium. Adherent cells were collected by trypsinization, resuspended in 1 ml Opti-MEM, combined with the collected medium in ice, and pelleted by centrifugation at 600 g for 3 min at 4°C. The cell pellet was washed in ice-cold PBS, resuspended in 110 μl Lysis Buffer (20 mM Tris pH 7.5, 150 mM NaCl, 1 mM EDTA pH 8.0, 10 mM NEM, 1%Triton X-100 supplemented with PIC) and transferred to a fresh 1.5 ml reaction tube on ice. After 15 min, the cell lysates were centrifuged at 600 g for 5 min at 4°C to pellet nuclei and generate a post-nuclear supernatant (PNS). The PNS was transferred to a fresh tube, mixed with 5x SDS-sample buffer (300 mM Tris, pH 6.8, 10% (w/v) SDS, 50% (v/v) glycerol, 10% (v/v) β-mercaptoethanol and 0.025% (w/v) bromophenol blue), heated for 5 min at 95°C and then subjected to SDS PAGE and immunoblot analysis.

### Data processing and statistical analysis

Microscopy images were processed using Fiji (ImageJ) software (National Institute of Health). Linescan analysis was performed on unprocessed images with the arrow tool in Fiji (width 5). Intensities were measured in each channel simultaneously at the same position. The obtained values were normalized by setting the maximum value to 100%. Pearson’s correlation coefficient of organellar markers with cgMito-cCer_6_ was calculated using the ImageJ plugin JACoP (39). Images of immunoblots and TLC plates were processed using ImageLab software (Version 6.0.1; BioRad). For quantification, intensities of bands were measured by using the volume tool and placing a box over the respective bands. It was ensured that rectangles over bands derived from the same blot/TLC plate always had the same area. For further calculations, the Adj. Vol. (Int) was used. For quantification of uptake of cgMito-cCer_6_, the values obtained at each timepoint were averaged and the highest mean value across was defined as 100%. The other time points, as well as the individual data points were expressed as a fraction of this maximum by dividing each value by the maximum and multiplying by 100. For calculation of PARP1 and Casp9 cleavage the following formula was used:

$$Relative\;Intensity=\frac{{Intensity}_{cleaved}}{{Intensity}_{cleaved}+{Intensity}_{uncleaved}}\times 100$$

Statistical significance was determined by performing an unpaired two-tailed *t* test in Graphpad Prism Software (Version 8.0.1). All graphs were plotted using Graphpad Prism Software.

## Results

### Chemical synthesis of mitochondria-targeted photocaged C_6_-ceramide

**Fig. 1:**
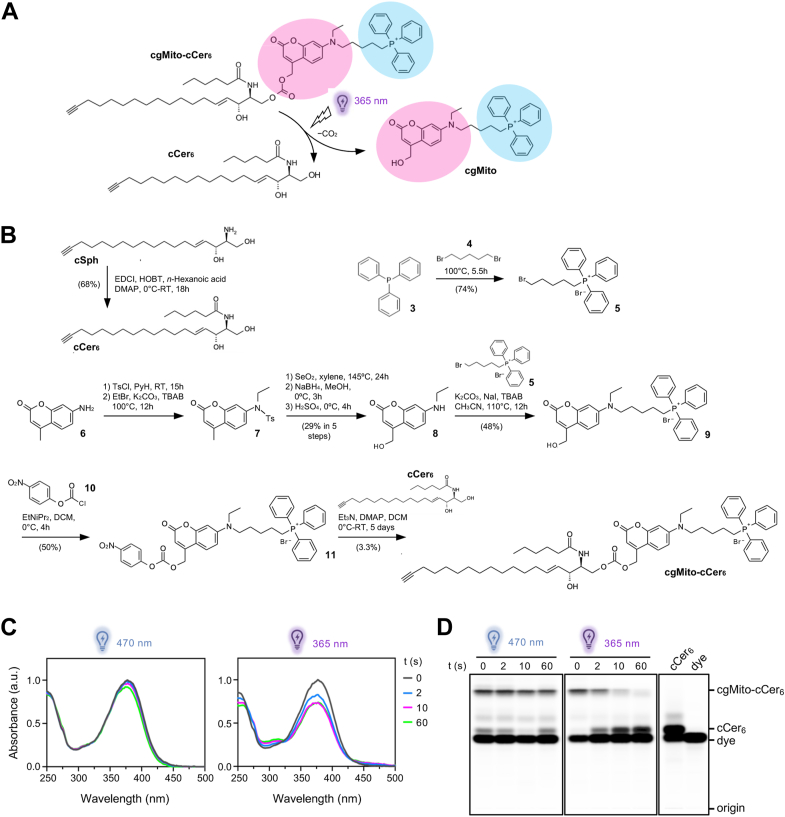
Design and synthesis of mitochondria-targeted photocaged and clickable C_6_-ceramide. A: Chemical structure of cgMito-cCer_6_, featuring a coumarin-based photocage (*magenta*) functionalized with a mitochondria-targeting triphenylphosphonium group (*blue*) and a clickable alkyne group (*green*). Irradiation with UV-light (365 nm) induces removal of the photocage (cgMito) and release of the clickable short-chain ceramide (cCer_6_). B: Chemical synthesis of cgMito-cCer_6_. See main text for details. C: UV-Vis absorption spectra of cgMito-cCer_6_ (16 μM in MeOH) after illumination for 0, 2, 10 and 60 s with blue (470 nm) or UV-light (365 nm). D: cgMito-cCer_6_ (50 μM in MeOH) was illuminated with blue (470 nm) or UV-light (365 nm) for 0, 2, 10 and 60 s. After illumination the alkyne moiety was click reacted with Alexa647-azide and the samples were analyzed by TLC.

We designed and synthesized *c*a*g*ed, *mito*chondria-targeted and *c*lickable-C_*6*_-*cer*amide (cgMito-cCer_6_) (Fig. 1A). A short-chain (C_6_-*N*-acyl) ceramide analog was synthesized because of its increased cell-permeability and since short-chain ceramide analogs have been shown to trigger apoptosis more effectively than their long-chain counterparts (40, 41), presumably owing to their more efficient cellular uptake and faster intracellular distribution. A coumarin-based photocage was attached to the hydroxyl group in position 1 of the sphingosine backbone to suppress the biological activity of the ceramide until its release by a flash of UV-light. As the coumarin moiety functions as a fluorophore, the photocaged compound can be readily visualized by fluorescence microscopy and thin-layer chromatography (TLC). For mitochondrial targeting, the photocage was functionalized with a triphenylphosphonium (TPP) group since lipids like arachidonic and sphingosine with TPP-modified photocages were previously shown to be effectively delivered to mitochondria (32, 35). The photocaged C_6_-ceramide carries a terminal alkyne in the sphingosine backbone for copper-catalyzed azide-alkyne cycloaddition (CuAAC) with an azide-containing fluorophore. This feature enables the detection of the ceramide moiety following its photorelease.

The construction of building blocks for the synthesis of cgMito-cCer_6_ (Fig. 1B) started with acylation of *c*lickable *sph*ingosine (cSph) 1, a compound that we previously reported on (42), with *n*-hexanoic acid in the presence of EDC/HOBt. This reaction afforded cCer_6_ (2) in very good yield. In parallel, triphenylphosphine 3 and 1,5-dibromopentane 4 were used to synthesize (5-bromopentyl)triphenylphosphonium bromide 5 as previously described (43). Starting from 7-amino-4-methylcoumarin 6, the synthesis of the coumarin cage 7-ethylamino-4-hydroxymethylcoumarin 8 was realized in 5 linear steps with an overall yield of 29%, as previously described (44, 45). *N*-alkylation of 7-ethylamino-4-hydroxymethylcoumarin 8 with 5-bromopentyl triphenylphosphonium bromide 5 in the presence of excess equivalent of potassium carbonate, sodium iodide and TBAB (cat.) in acetonitrile medium afforded the required triphenylphosphonium photocage 9 in good yield. The photocage was activated using *p*-nitrophenyl chloroformate 10, yielding reactive intermediate 11, which readily conjugated with cCer_6_ 2 to create cgMito-cCer_6_. Further details on the chemical synthesis of cgMito-cCer_6_ can be found in the Supporting Information.

### Photophysical properties and uncaging efficiency of cgMito-cCer_6_

To evaluate the photophysical properties and uncaging efficiency of cgMito-cCer_6_, we recorded UV-VIS spectra after the compound was illuminated either with blue (470 nm) or UV-A (365 nm) light for different periods of time (Fig. 1C). Under blue-light conditions, no notable change in UV-VIS spectra was observed, indicating photostability. However, UV-illuminated samples demonstrated a rapid decrease in the maximum absorbance already after 2 s of illumination. After 10 s, this decrease was even more pronounced, but this effect did not continue after prolonged illumination for 60 s. We assumed that these changes in absorbance were due to uncaging and photorelease of cCer_6_. To confirm this, cgMito-cCer_6_ was subjected to the same light regime followed by derivatization of its alkyne group with a fluorophore and TLC analysis (Fig. 1D). Again, while under blue-light conditions cgMito-cCer_6_ remained stable, UV-A-treatment led to loss of the cgMito-cCer_6_ band over time. Simultaneously, a band at the height of the lipid marker cCer_6_ gradually accumulated, demonstrating photorelease of short-chain ceramide. Uncaging was almost complete after 10 s of UV-illumination, which is in line with the results obtained by UV-Vis spectroscopy (Fig. 1C).

### cgMito-cCer_6_ but not cgMito is readily taken up by cells

**Fig. 2:**
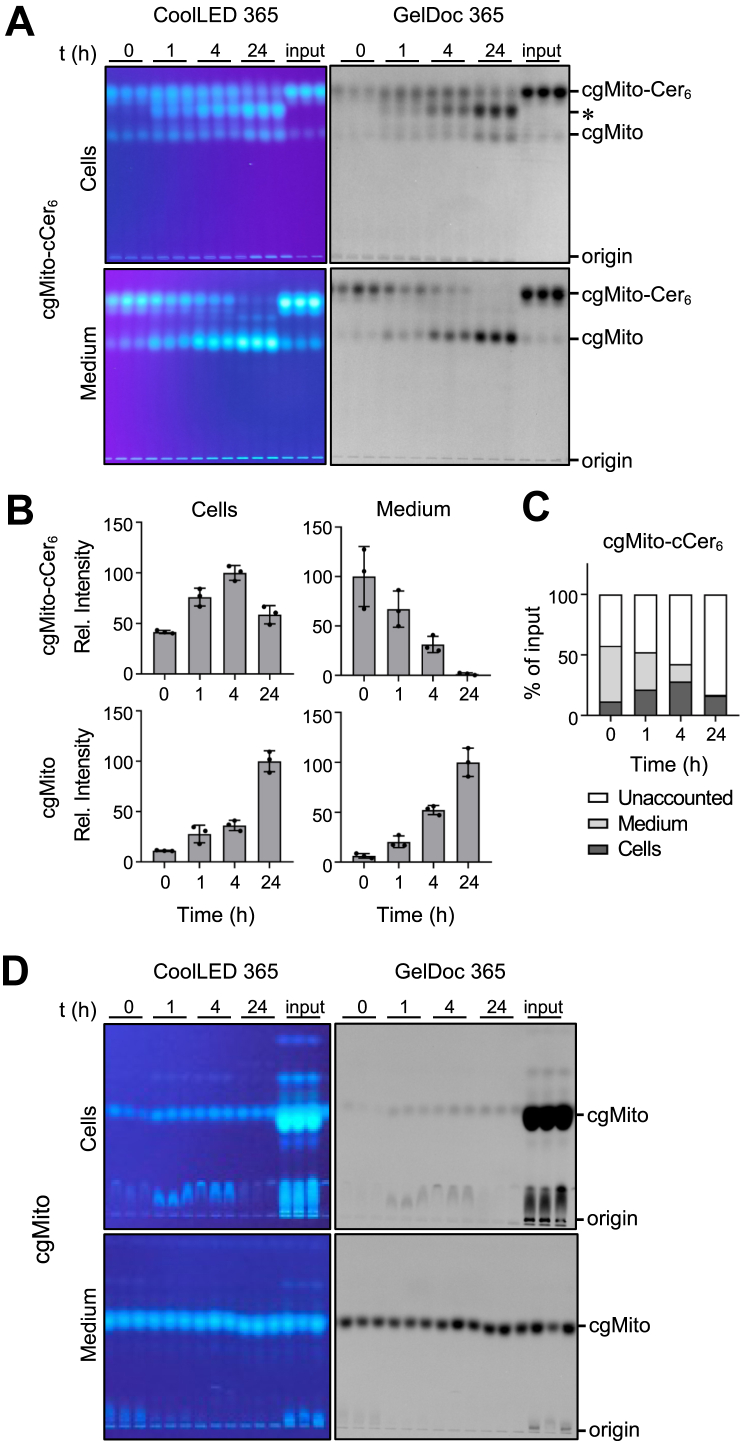
Cellular uptake and stability of cgMito-cCer_6_. A: HeLa cells were incubated for up to 24 h in medium containing cgMito-cCer_6_ (10 μM). After the indicated times, medium and cells were harvested and subjected to lipid extraction and TLC analysis. Fluorescence of the coumarin photocage was detected upon illumination at 365 nm using a CoolLed or GelDoc device. B: Quantification of relative cgMito-cCer_6_ uptake at the indicated timepoints in HeLa cells. Data shown are mean values ± SD from three independent biological replicates (n = 3). C: Quantification of cgMito-cCer_6_ uptake in HeLa cells as a percentage of total input. Data shown are mean values from three independent biological replicates (n = 3). D: HeLa cells were incubated for up to 24 h in medium containing cgMito (10 μM). After the indicated times, medium and cells were harvested and subjected to lipid extraction and TLC analysis. Fluorescence of the coumarin photocage was detected as in (A). A 10-fold higher amount of cell-derived lipid extracts was analyzed compared to the medium-derived lipid extract.

We next examined whether cgMito-cCer_6_ is effectively taken up by cells. To this end, we fed HeLa cells 10 μM cgMito-cCer_6_ in the dark and evaluated the amount which was cell-associated or retained in the medium after different incubation periods via detection of the photocage fluorescence by TLC-analysis (Fig. 2A, B). Over time, an increase in cell-associated cgMito-cCer_6_ was observed, with a maximum relative uptake at 4 h. At 24 h, the amount of cgMito-cCer_6_ decreased again. Notably, a band of unknown origin (∗) with a slower migration on TLC appeared after 1 h and gradually accumulated in the cells over the course of the incubation. As this product was not present in the medium, it appears likely that its formation is due to metabolic conversion of cgMito-cCer_6_. However, the identity of the cgMito-cCer_6_-derived product and the enzyme(s) responsible for its formation remains to be established. In parallel, a continuous decrease in cgMito-cCer_6_ was observed in the medium, with no compound left after 24 h. Simultaneously, an increase over time in free photocage (cgMito) was noted in both cells and medium. This light-independent decomposition of cgMito-cCer_6_ was more apparent in the medium than in cells. On average 11%, 21%, 28% and 16% of the input was detectable in cells after 0, 1, 4 or 24 h respectively, while 46%, 30%, 14% and 0.5% was retained in the medium at the same time (Fig. 2C). A major fraction of the input, corresponding to 42%, 47%, 57% and 82% after 0, 1, 4 and 24 h, respectively, was not retrievable from either the cells or the medium. This loss may be attributed to compound adhering to the culture dish combined with ongoing decomposition over time. To test whether decomposition of cgMito-cCer_6_ in the medium would lead to a gradual increase in cellular uptake of cgMito, we fed HeLa cells the free photocage for 0, 1, 4 and 24 h (Fig. 2D). However, only a very minor fraction of the input cgMito became cell-associated over the course of the incubation, while the bulk remained in the medium. This effect was so pronounced that a 10-fold higher amount of the cellular lipid extract had to be applied for TLC analysis to detect the compound.

Collectively, these results demonstrate that cgMito-cCer_6_ is efficiently taken up by HeLa cells and remains mostly stable for up to 1 h. Over a longer incubation period, cells gradually accumulated a cgMito-cCer_6_-derived product that is likely due to metabolic conversion of the intact compound. Furthermore, only a very minor fraction of cgMito released during a light-independent decomposition of cgMito-cCer_6_ in the medium was taken up by the cells. To minimize decomposition and metabolic conversion of the compound, cells were fed cgMito-cCer_6_ for a maximum of 1 h in all subsequent experiments.

### cgMito-cCer_6_ is exclusively targeted to mitochondria

**Fig. 3:**
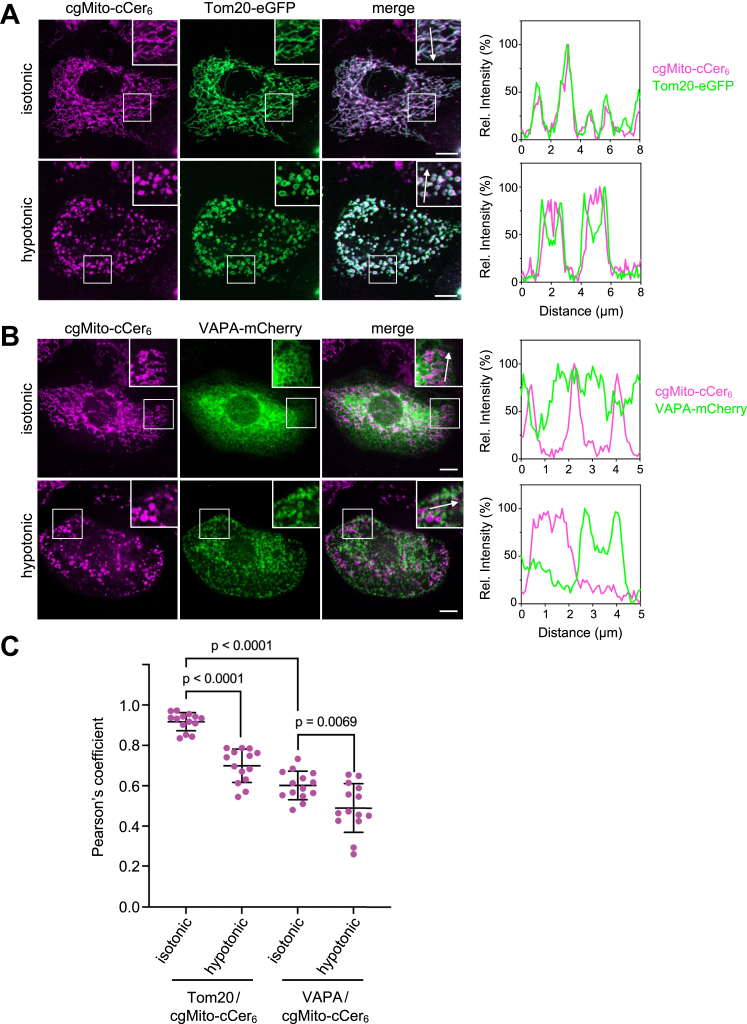
cgMito-cCer_6_ is specifically targeted to mitochondria. A: HeLa cells transfected with Tom20-eGFP were incubated with cgMito-cCer_6_ (5 μM, 1 h), washed, and then first imaged in isotonic (100% Opti-MEM) and afterwards in hypotonic medium (1% Opti-MEM, 5 min incubation) by spinning disk microscopy. Line scans show degree of overlap between cgMito-cCer_6_ and Tom20 along the path of the arrow shown in the zoom-in. Scale bar, 10 μm. B: HeLa cells transfected with VAPA-mCherry were incubated with cgMito-cCer_6_ (5 μM, 1h), treated as in (A) and then imaged by spinning disk microscopy. Line scans show degree of overlap between cgMito-cCer_6_ and VAPA signals along the path of the arrows shown in the zoom-ins. Scale bar, 10 μm. (C) Pearson’s correlation co-efficient analysis between cgMito-cCer_6_ with Tom20-eGFP (n = 14 cells) or VAPA-mCherry (n = 14 cells). The same cells were analyzed before and after hypotonic swelling. Statistical significance was assessed using an unpaired two-tailed *t* test.

To determine the subcellular distribution of cgMito-cCer_6_ taken up by the cells, HeLa cells were fed cgMito-cCer_6_ for 1 h, washed, and then visualized by live cell spinning disk microscopy. As shown in Figure 3A, cgMito-cCer_6_ displayed extensive co-localization with eGFP-tagged mitochondrial outer membrane (MOM) protein Tom20, demonstrating efficient targeting to mitochondria. Additionally, to monitor the sub-organellar distribution of cgMito-cCer_6_, cells were subjected to hypotonic swelling as described before (46, 47). After incubation for 5 min in hypotonic medium, the mitochondrial tubular network transformed into numerous micrometer-sized vesicles. While the limiting membrane of the mitochondria-derived microvesicles was extensively labelled with Tom20-eGFP, the cgMito-cCer_6_ signal was primarily found in the vesicular lumen, indicating delivery of the compound to the inner mitochondrial membrane (IMM) or matrix (Fig. 3A, C).

Since the ER represents the most abundant organellar membrane (48) and serves as the cellular site of *de novo* ceramide synthesis (49), we also performed co-localization experiments in cells expressing mCherry-tagged ER-resident membrane protein VAPA and tested whether some of the compound is mislocalized to the ER. However, no co-localization was observed between VAPA-mCherry and cgMito-cCer_6_ (Fig. 3B, C). Moreover, hypotonic swelling led to a complete segregation of VAPA-positive microvesicles from cgMito-cCer_6_-positive microvesicles, further confirming selective targeting of the compound to mitochondria.

**Fig. 4:**
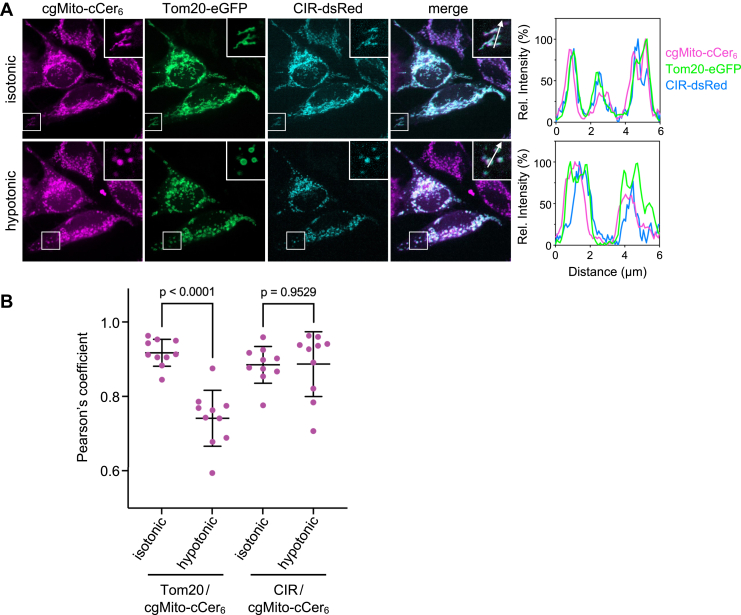
cgMito-cCer_6_ accumulates in the IMM/matrix. A: HeLa cells co-transfected with Tom20-eGFP and CIR-dsRed were incubated with cgMito-cCer_6_ (5 μM, 1 h). After washing, cells were first imaged in isotonic (100% Opti-MEM) and afterwards in hypotonic medium (1% Opti-MEM, 5 min incubation) by spinning disk microscopy. Line scans show degree of overlap between Tom20, CIR and cgMito-cCer_6_ signals along the path of the arrows shown in the zoom-ins. Scale bar, 10 μm. B: Pearson’s correlation co-efficient analysis between cgMito-cCer_6_ with Tom20-eGFP or CIR-dsRed. The same cells were analyzed before and after hypotonic swelling. Statistical significance was assessed using an unpaired two-tailed *t* test. (n = 10 cells).

Cellular uptake of TPP-conjugated lipophilic molecules from the extracellular environment is facilitated by the plasma membrane potential. From the cytoplasm, such compounds typically accumulate on the matrix surface of the IMM driven by the membrane potential across the IMM (50). Thus, we wanted to test whether the presence of cgMito-cCer_6_ inside mitochondria-derived microvesicles (Fig. 3A) reflected delivery of the compound to the IMM/matrix. To this end, we co-expressed Tom20-eGFP with dsRed-tagged NDUFS3 (CIR), a peripheral IMM protein and 30 kDa subunit of Complex-I of the respiratory chain (51). Under isotonic conditions, both protein markers exhibited extensive co-localization with cgMito-cCer_6_ (Fig. 4A). However, after hypotonic swelling, the signals of Tom20 and CIR segregated, with Tom20 staining the limiting membrane of the mitochondrial microvesicles and CIR displaying a luminal distribution. As before, cgMito-cCer_6_ is localized to the CIR-positive lumen of these vesicles. This was further supported by reduced colocalization of cgMito-cCer_6_ with Tom20-eGFP, while co-localization with CIR-dsRed remained high (Fig. 4B), indicating its accumulation in the IMM/matrix.

Based on these results, we conclude that cgMito-cCer_6_ is efficiently and exclusively targeted to mitochondria.

### Uncaging of cgMito-cCer_6_ induces mitochondrial apoptosis

**Fig. 5:**
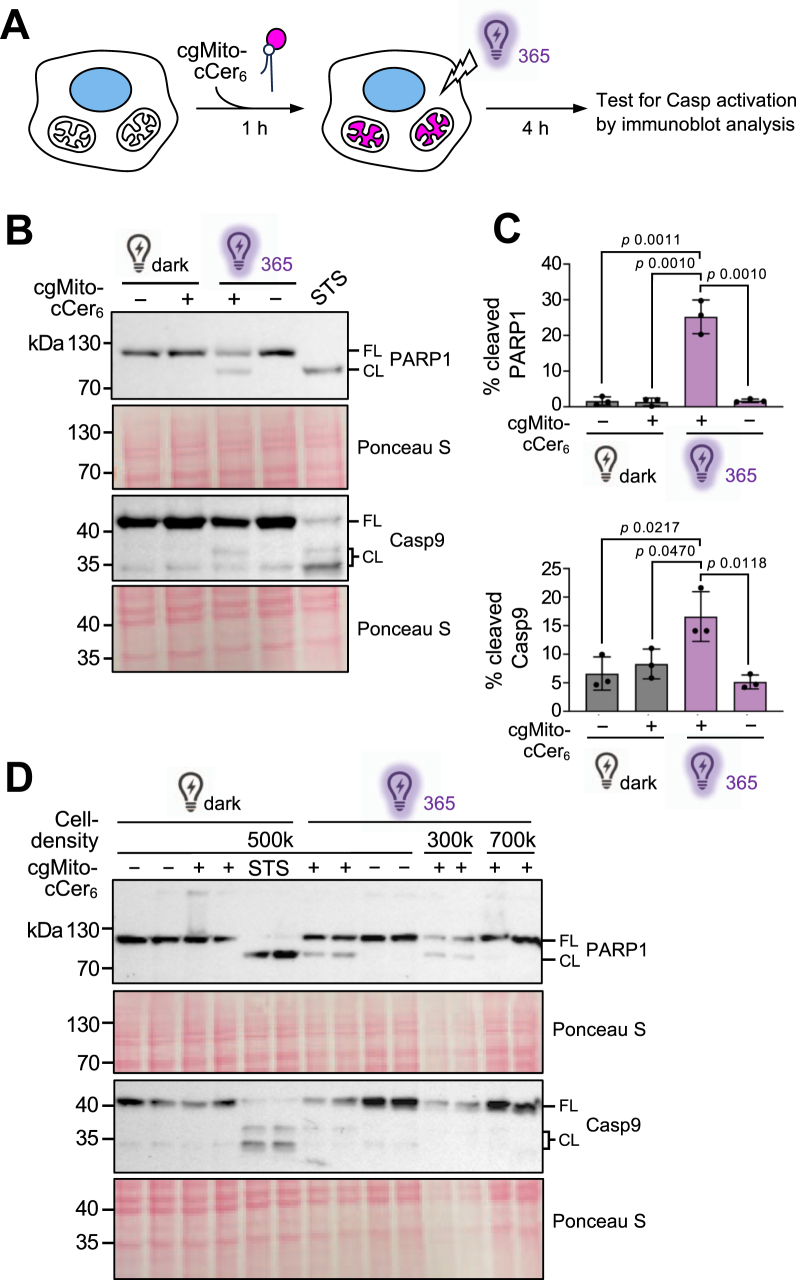
cgMito-cCer_6_ induces mitochondrial apoptosis. A: Schematic outline of the workflow to test whether photorelease of ceramide from cgMito-cCer_6_ inside mitochondria triggers apoptosis. B: HeLa cells were seeded at 500k per 9.6 cm^2^ well, grown overnight, and then incubated either with EtOH as vehicle control (−) or 10 μM cgMito-cCer_6_ for 1 h. Next, cells were washed, UV-irradiated for 30 s or kept in the dark, incubated for 4 h, and probed for cleavage of PARP1 and Casp9 by immunoblot analysis. Ponceau staining served as loading control. C: Quantification of the relative level of PARP1 and Casp9 cleavage in cells treated as in (B). Data shown are mean values ± SD from three independent biological replicates (n = 3). Statistical significance was assessed using an unpaired two-tailed *t* test. D: Cells seeded at the indicated densities were treated as in (B) and probed for cleavage of PARP1 and Casp9 by immunoblot analysis. Ponceau staining served as loading control. FL, full length; CL, cleaved.

After confirming efficient mitochondrial delivery of cgMito-cCer_6_, we set out to assess whether photorelease of ceramide inside mitochondria triggers apoptosis. To this end, HeLa cells were incubated with or without cgMito-cCer_6_ for 1 h, washed, and subjected to UV-illumination or kept in the dark (Fig. 5A). Following 4 h of incubation, cells were lysed and probed for cleavage of Poly-ADP-Ribose Polymerase 1 (PARP-1), a well-known marker of caspase-dependent apoptosis (52), and cleavage of caspase 9 (Casp9), an initiator caspase with a central role in the mitochondrial apoptotic pathway (53). Cells treated with staurosporin (STS), a widely-used chemical inducer of mitochondrial apoptosis, served as positive control. As expected, STS treatment led to robust cleavage of PARP1 and Casp9. Cells fed with cgMito-cCer_6_ and exposed to UV-light displayed cleavage of both PARP1 and Casp9 (Fig. 5B, C). On average, 25% of total PARP1 and 17% of total Casp9 underwent cleavage. In contrast, cells exposed to UV-light in the absence of cgMito-cCer_6_ or cgMito-cCer_6_-fed cells that were kept in the dark did not display any PARP1 or Casp9 cleavage, at least not beyond the extend observed in cells incubated in the dark in the absence of the compound (Fig. 5B, C). From this, we conclude that photorelease of ceramide inside mitochondria triggers apoptosis. Interestingly, the ability of UV-irradiated cgMito-cCer_6_, to trigger PARP1 and Casp9 cleavage appeared to depend on cell density (Fig. 5D). Cells seeded at lower densities (∼300k or ∼500k cells per 9.6 cm^2^) were significantly more sensitive to apoptosis induced by uncaging of mitochondrial cgMito-cCer_6_ than cells seeded at higher densities (∼700k cells per 9.6 cm^2^). This is in line with previous studies, which revealed that cells at higher densities display an enhanced resistance against apoptotic stimuli (54, 55, 56).

Collectively, these results demonstrate the suitability of cgMito-cCer_6_ as a novel tool to place ceramide-induced mitochondrial apoptosis under optical control.

## Discussion

We previously showed that diverting CERT-mediated ceramide transport to mitochondria induces BAX-dependent apoptosis (22, 23). Here, we demonstrate that photorelease of ceramides inside mitochondria initiates cleavage of Casp9 and PARP1, signifying activation of the intrinsic apoptotic pathway. These findings reinforce the notion that ceramides can act directly on mitochondria to commit cells to death and establish mitochondria-targeted photocaged ceramides as novel tools to dissect the mechanism underlying ceramide-mediated apoptosis with the unmatched spatiotemporal precision of light. Our previous work revealed that CERT variants equipped with an OMM anchor enable a gradual rise in mitochondrial ceramides, with the amount of ceramides supplied being limited by the rate of de-novo ceramide synthesis in the ER (22, 23). Mitochondria-targeted photocaged ceramides provide a more acute method for manipulating mitochondrial ceramide levels by enabling instant release of bioactive lipids from the entire organellar pool of inactive precursors via a short flash of light. As a result, cells have less time to mount an adaptive response, which would make it harder to deconvolute the sequence of events through which ceramides commit cells to death. Moreover, the application of photocaged ceramides bypasses the need of genetic engineering and circumvents potential side effects associated with diverting biosynthetic ceramide flows. Although photocaged ceramides of varying chain lengths have been previously reported (57, 58, 59), the present study constitutes the first example of a photocaged ceramide specifically targeted to mitochondria to investigate its apoptogenic activity.

Given the markedly enhanced cellular uptake of short-chain ceramides compared to their long-chain counterparts (40, 41), we designed a photocaged C_6_-*N*-acyl ceramide analog, cgMito-cCer_6_. We found that cgMito-cCer_6_ is readily taken up by cells, where it remained mostly stable for at least 1 h under physiological conditions. Furthermore, cgMito-cCer_6_ accumulated selectively in mitochondria, precisely within the IMM/matrix. This targeting was highly specific, as evidenced by the lack of co-localization with the ER, the largest membrane-bound cellular organelle. This specificity ruled out off-target effects caused by ceramides photoreleased in other subcellular compartments. Uncaging of cgMito-cCer_6_ in mitochondria resulted in the proteolytic cleavage of the apoptotic markers PARP1 and Casp9, consistent with activation of the intrinsic, mitochondrial pathway of apoptosis. In contrast, cells kept in the dark or UV irradiated in the absence of cgMito-cCer_6_ showed no enhanced PARP1 or Casp9 cleavage, indicating that apoptosis induction was strictly dependent on photorelease of ceramide inside mitochondria.

While the apoptogenic activity of cgMito-cCer_6_ was effectively suppressed in the absence of UV irradiation, a gradual accumulation of a cgMito-cCer_6_-derived product inside dark-adapted cells suggested that the intact compound was still recognized by the cell’s lipid metabolic machinery. As this product migrated more slowly on TLC than cgMito-cCer_6_, we postulate that its formation may be due to the removal of the C_6_-acyl chain by a ceramidase. Although information on mitochondrial ceramidase activity is limited, there are reports on neutral-ceramidase ASAH2 displaying ceramidase activity in mitochondria (60, 61, 62). This raised the question of whether effects observed upon UV-irradiation of cgMito-cCer_6_-fed cells may be caused by photorelease of sphingosine. However, Feng *et al.* (34) previously developed a mitochondria-targeted caged sphingosine and showed that sphingosine photoreleased inside mitochondria is rapidly (within min) converted into sphingosine-1-phosphate (S1P), a signaling lipid that stimulates cell growth and suppresses apoptotic cell death (19, 63). Moreover, photorelease of sphingosine in mitochondria did not lead to any signs of reduced cell viability (35). From this, we conclude that the induction of apoptosis observed in cgMito-cCer_6_-fed cells upon UV-irradiation is a direct consequence of mitochondrial photorelease of cCer_6_.

Although cgMito-cCer_6_ inside cells is subject to metabolic turnover, its photocage stayed rather stably attached to the lipid moiety over a prolonged period of time. In contrast, cgMito-cCer_6_ present in the culture medium displayed a light-independent hydrolysis of the photocage, with no intact compound left after 24 h of incubation. We hypothesize that this may be due to the presence of high concentrations of free amino acids, in particular lysine and cysteine, which could undergo an SN2 reaction with the activated carbonyl functionality present in cgMito-cCer_6_. This nucleophilic substitution would cause the release of free photocage and formation of a cCer_6_-carbamate or cCer_6_-thiocarbonate conjugate. Arguably, in a cellular context, this reaction might be less viable, as the concentration of free amino acids inside mammalian cells is substantially lower than that in culture medium (64). Additionally, upon arrival in the IMM, the carbonate linker of cgMito-cCer_6_ might be largely protected from such nucleophilic substitution, explaining the relative stability of the compound upon cellular uptake. Despite an ongoing release of free photocage in the medium, no considerable increase in the amount of intracellular cgMito could be detected. This observation aligns with the notion that lipidation of small molecules significantly enhances their cellular uptake (65). Thus, the ceramide conjugation appears to play a crucial role in enabling efficient delivery of cgMito-cCer_6_ inside cells. While previous studies monitored the intracellular stability of photocaged lipids (33, 34, 66), our results emphasize that their stability in the extracellular medium should also be carefully assessed.

Our previous work revealed that ceramides exert their apoptogenic activity in the OMM at least in part via VDAC2, which harbors a charged membrane-buried glutamate (E84) that mediates direct contacts with the ceramide headgroup (24). The same glutamate residue is also required for mitochondrial recruitment of HKI (26). VDAC-bound hexokinases play a pivotal role in tumor progression (67). In addition, HKI binding to VDAC2 promotes retro-translocation of pro-apoptotic BCL2 family proteins from the OMM into the cytosol, thereby attenuating apoptotic signaling (67, 68, 69). The notion that HKI and ceramides share a common binding site on VDACs points at a potential mechanism by which ceramides exert their tumor suppressor activities. Future studies should reveal whether ceramides compete directly with HKI for binding to the charged glutamate on the VDAC channel wall and whether their anti-neoplastic activity is linked to a displacement of HKI from mitochondria. We envision that these efforts will benefit from cgMito-cCer_6_ as a novel tool to acutely manipulate mitochondrial ceramide pools. Although the light-induced release of ceramides from cgMito-cCer_6_ occurs selectively in the IMM, we expect short-chain ceramides liberated in the IMM to readily diffuse to the OMM. Consequently, it will be of primary interest to investigate whether VDAC2 participates in the mechanism by which uncaging of cgMito-cCer_6_ triggers apoptosis.

## Data availability

All data generated in this study are included in the manuscript and Supplementary Information file.

## Supplemental data

This article contains supplemental data (42, 43, 44, 45, 70, 71, 72, 73, 74, 75, 76, 77).

## Conflict of interests

The authors declare that they have no conflicts of interest with the contents of this article.
