# Supplementary material for: Mitochondria-specific photorelease of ceramide induces apoptosis
Source: J Lipid Res. 2025 Sep 19;66(11):100907. doi: 10.1016/j.jlr.2025.100907 (PMC12590141; doi:10.1016/j.jlr.2025.100907)
Supplement: Supplemental Data [file mmc1.pdf]

SUPPLEMENTAL INFORMATION

Mitochondria-specific photorelease of ceramide induces apoptosis

Christian Schröer[1,2], Matthijs Kol[1,2], Anna Koch[1,2], Emely Döffinger[1,2], Murali Anamalai[1,2], Joost C.M. Holthuis[1,2]

[1]Division of Molecular Cell Biology, Department of Biology/Chemistry, Osnabrück University, 49076 Osnabrück, Germany.
[2]Center for Cellular Nanoanalytics, Osnabrück University, Artilleriestraße 77, 49076 Osnabrück, Germany.

1. Chemical Synthesis of cSph and cgMito-$cCer_6$

1.1 General Considerations

Starting compounds and solvents were purchased from the relevant suppliers and used as received. Reactions were carried out under argon in a dry Schlenk flask, protected from light if necessary. Unstabilized dry grad solvents, packed under nitrogen, were used for the reactions: dichloromethane (CH_2Cl_2, SeccoSolv, Merck), acetonitrile (Sigma- Aldrich), diethylether (Et_2O, SeccoSolv, Merck), methanol (MeOH, Fisher), dimethylformamide (DMF, Roth) and tetrahydrofuran (THF, Sigma). Column chromatography: silica gel 60 (SiO_2 ; Merck, Germany) and Combi-Flash flash chromatography system with prepacked silica cartridges. TLC: aluminium plates, SiO_2 60 F254, 0.2-mm layer (Merck, Germany). NMR Spectra: AMX-500 spectrometer (Bruker, DHelvetica Rheinstetten); ^{1}H: 500.14, ^{13}C: 125.76 and ^{31}P: 101.3 MHz; d in ppm rel. to the signals of the residual protons of $CHCl_3$ (δ =7.26 ppm) in $CDCl_3$ or CH_3OH (δ = 3.35 ppm) in CD_3OD, carbons of $CDCl_3$ (δ =77.00 ppm) or CD_3OD (δ = 49.3 ppm) and external 85% H_3PO_4 for ^{31}P (δ = 0 ppm); J in Hz. ESI-MS: Bruker Daltronics Esquire HCT instrument (Bruker Daltonics, Bremen); ionization was performed with a 2% aq. HCO_2H soln. ESI-MS spectra were measured on a Hitachi M-4100 Tandem Mass Spectrometer or a JEOL JMS-T100LC spectrometer. Commonly used abbreviations are applied to refer to the following reagents and structural fragments: DMAP (4-(dimethylamino)-pyridin), HOBT (1-hydroxybenzotriazol), EDCI (1-ethyl-3-(3-dimethylaminopropyl)carbodiimide), TBAB (tetrabutylammonium bromide), HMPTA (hexamethylphosphoric triamide), TBAF (tetrabutylammonium fluoride), TMS (trimethylsilyl), TBDMS (*tert*-butyldimethylsilyl), Boc (*tert*-butyloxycarbonyl).

1.2 Compound Synthesis and Characterization

1.2.1 Synthesis of clickable Sphingosine (cSph)

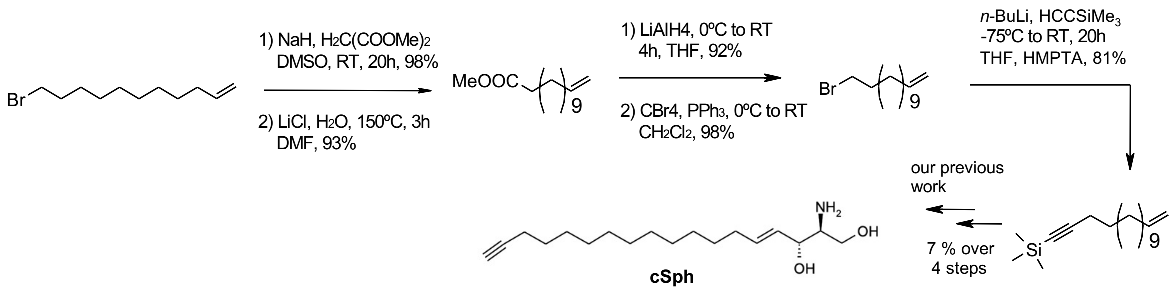

Scheme 1. Scheme for the synthesis of **cSph**.

Clickable sphingosine (**cSph**) was synthesized according to Scheme 1. The head group was prepared from Boc-L-serine (Novabiochem) in 4 steps with an overall yield of 52% as previously described (Yamamoto *et al.,* 2006; Mina *et al.,* 2011). To obtain the C_{15}-olefin chain, a new efficient scheme with five steps was developed to gain an overall high yield of 66%. The procedure involved the alkylation of dimethyl-malonate with 11-bromundecene followed by decarbomethoxylation and reduction of the resulting monoester to tridec-12-en-1-ol. The latter was converted to 13-bromotridec-1-ene by *Appel* bromination followed by alkylation with lithium rimethylsilylacetylide to the desired olefin chain. The C_{15}-olefin chain was combined with the head group by cross-metathesis using the Grubbs cat 2nd generation. Subsequent removal of the three protecting groups led to the highly pure **cSph** with a yield of 10% yield. Trimethylpentadec-14-en-1-ynysilane, one of the cross-metathesis partners in the synthesis of **cSph**, was prepared by an improved method, as described below.

2-Undec-10-enylmalonic acid dimethyl ester (S1)

A solution of dimethyl malonate (2.64 g, 0.02 mol) in dimethyl sulfoxide (4 ml) was added dropwise to a suspension of sodium hydride (530 mg, 0.022 mol, Sigma-Aldrich, 60% suspension in oil, washed with pentane under argon before reaction) in dimethyl sulfoxide (10 ml) at RT and stirred for 20 min until a clear yellowish solution formed. A solution of 11-bromoundec-1-ene (2.33 g, 0.01 mol) in dimethyl sulfoxide (5 ml) was added dropwise during 5 min and the reaction mixture was stirred at room temperature overnight. The reaction mixture was treated with water, extracted with diethyl ether (3 x 60 ml), extracts were washed with water (3 x 30 ml), dried (Na_2SO_4) and concentrated to give almost quantitative yield of the alkylated malonate, which was used for the next step without further purification (dialkylated malonic ester was detected in traces, but not separated at this step). For analytical purpose, the monoalkylated malonate **S1** was purified by chromatography on silica gel (eluted with a mixture petroleum ether – dichloromethane 1:1, v/v).

TLC (petroleum ether – dichloromethane 1:1, v/v): R_f 0.25 (monoalkylmalonate **S1**), 0.5 (dialkylmalonate). Colourless, mobile liquid.

^{1}H-NMR (500 MHz, $CDCl_3$): δ 5.85-5.77 (*m*, 1H, HC=), 4.99 (*dd*, 1H, *J* = 17.5, 2.0, =C$\underline{H}$H), 4.93 (*dd*, 1H, *J* = 10.5, 1.0, =CH$\underline{H}$), 3.73 (*s*, 6H, $COOCH_3$), 3.35 (*t*, 1H, *J* = 7.5, $\underline{H}C(COO)_2$), 2.04 (*q*, 2H, *J* = 7.5, H_2CC=), 1.89 (*q*, 2H, *J* = 7.0, $\underline{H_2}CCHCOO$), 1.37 (*quintet*, 2H, *J* = 7.0, CH_2), 1.30-1.26 (*m*, 12H, CH_2).

^{13}C-NMR (126 MHz, $CDCl_3$): δ 169.93 (OC=O), 139.18 (=CH), 114.07 (=CH_2), 52.34 (OCH_3), 51.73 (H$\underline{C}$COO), 33.75 ($H_2\underline{C}C$=), 29.40 (CH_2), 29.38 (CH_2), 29.24 (CH_2), 29.14 (CH_2), 29.06 (CH_2), 28.90 (CH_2), 28.84 (CH_2), 27.03 (CH_2).

^{1}H-NMR spectrum is in a good agreement with that reported (Ohwada, *et al.*, 2004).

Tridec-12-enoic acid methyl ester (S2)

A mixture of the monoalkylmalonate **S1** from the previous step (2.8 g, 0.01 mol) and lithium chloride (0.46 g, 0.011 mol, Sigma-Aldrich) was heated at 150 °C (bath temperature) for 3 h in a mixture of dimethylformamide and water (10 ml, 50:1, v/v) until the calculated volume of carbon dioxide was completely released. The reaction mixture was quenched with water (30 ml), extracted with diethyl ether (4 x 25 ml), extracts were washed with water (4 x 30 ml), dried (Na_2SO_4) and concentrated to give 2.07 g (92%) of the final product **S2**, as a mobile liquid, which was used on the next step without further purification (dialkylated ester was present in traces, but was not separated at this step). For analytical purpose, the methyl ester **S2** was purified by chromatography on silica gel (eluted with mixture petroleum ether – dichloromethane 2:1, v/v).

TLC (petroleum ether – dichloromethane 1:1, v/v): R_f 0.5 (monoalkyl-), 0.69 (dialkyl-). Colourless, mobile liquid.

^{1}H-NMR (500 MHz, $CDCl_3$): δ 5.87-5.79 (*m*, 1H, HC=), 5.00 (*dd*, 1H, *J* = 17.0, 1.5, =C$\underline{H}$H), 4.94 (*dd*, 1H, *J* = 10.0, 1.0, =CH$\underline{H}$), 3.68 (*s*, 3H, OCH_3), 2.31 (*t*, 2H, *J* = 7.5, H_2CCOO), 2.05 (*q*, 2H, *J* = 7.0, H_2CC=), 1.62 (*quintet*, 2H, *J* = 7.5, CH_2), 1.39 (*quintet*, 2H, *J* = 7.0, CH_2), 1.29 (*br.s,* 12H, CH_2).

^{13}C-NMR (126 MHz, $CDCl_3$): δ 174.26 (OC=O), 139.20 (=CH), 114.05 (=CH_2), 51.35 (OCH_3), 34.10 (H$\underline{C}$COO), 33.76 ($H_2\underline{C}C$=), 29.48 (CH_2), 29.42 (CH_2), 29.39 (CH_2), 29.20 (CH_2), 29.12 (CH_2), 29.09 (CH_2), 28.92 (CH_2), 24.94 (CH_2).

^{1}H and ^{13}C-NMR spectra are in agreement with those reported for the closest known C_{14} homologue (Gan, *et al.*, 2006).

Tridec-12-en-1-ol (S3)

A powder of lithium aluminum hydride (1.38 g, 36 mmol, Sigma-Aldrich) was added in portions under argon to a stirred pre-cooled (ice bath) solution of tridec-12-enoic acid methyl ester (**S2)** (2.06 g, 9 mmol) in 10 min (*caution: after the addition of the first portion it is important to wait for the reaction to begin!*). After 15 min the cooling bath was removed and the reaction mixture was stirred at room temperature overnight. Methanol (6 ml) was added to the pre-cooled (ice bath) reaction mixture dropwise over 4 min (slight foaming occurs) followed by the addition of water (2

ml) to decompose the excess of the reductant, the reaction mixture was diluted with diethyl ether (50 ml) and filter through a SiO_2 layer (2 cm), eluted with a mixture diethyl ether – methanol (200 ml, 10:1 v/v). Filtrate was concentrated and after chromatography over SiO_2 (eluted with gradient mixture petroleum ether – dichloromethane, from 9:1 to 1:2, v/v) tridec-12-en-1-ol (**S3)** (1.65 g, 91%) was obtained as a colourless liquid.

TLC (petroleum ether – dichloromethane, 1:2, v/v): R_f 0.15. Liquid.

^{1}H-NMR (500 MHz, $CDCl_3$): δ 5.87-5.79 (*m*, 1H, HC=), 5.01 (*dd*, 1H, *J* = 17.0, 1.5, =CHH), 4.94 (*dd*, 1H, *J* = 10.5, 1.0, =CHH), 3.65 (*t*, 2H, *J* = 7.0, OCH_2), 2.06 (*q*, 2H, *J* = 7.0, H_2CC=), 1.58 (*quintet*, 2H, *J* = 7.5, H_2CCH_2O), 1.38 (*quintet*, 2H, *J* = 7.0, CH_2), 1.29 (*br.s,* 12H, CH_2).

^{13}C-NMR (126 MHz, $CDCl_3$): δ 139.22 (=CH), 114.05 (=CH_2), 63.08 (OCH_2), 33.77 (H_2CC=), 32.81 (H_2CCH_2O), 29.56 (CH_2), 29.55 (CH_2), 29.53 (CH_2), 29.45 (CH_2), 29.40 (CH_2), 29.11 (CH_2), 28.93 (CH_2), 25.72 (CH_2).

^{1}H NMR spectrum is in a good agreement with that reported (Fenlon, *et al*., 2008).

13-Bromotridec-1-ene (S4)

A filtered solution of tetrabromomethane (4.96 g, 15 mmol, Merck) in dichloromethane (6 ml) was added dropwise in 2 min to a pre-cooled (ice bath) mixture of tridec-12-en-1-ol (**S3**) (1.65 g, 8.3 mmol) and triphenylphosphine (3.93 g, 15 mmol) in dichloromethane (20 ml) and stirred overnight. The reaction mixture was diluted with petroleum ether (30 ml), the resulting cloudy suspension was stirred for 15 min until the fine precipitate coagulated, this was filtered through a layer of SiO_2 (2 cm), washed with a mixture petroleum ether-dichloromethane (20 ml, 1:1) and concentrated. The residue was re-dissolved in petroleum ether (20 ml), the precipitate formed was separated, washed with petroleum ether, the filtrate was concentrated and after chromatography over SiO_2 (eluted with petroleum ether) 13-bromotridec-1-ene (**S4)** was obtained with a yield 2.13 g (98%) as a colourless liquid.

TLC (petroleum ether): R_f 0.72. Colourless liquid.

^{1}H-NMR (500 MHz, $CDCl_3$): δ 5.87-5.79 (*m*, 1H, HC=), 5.03-4.99 (*m*, 1H, =CHH), 4.96-4.94 (*m*, 1H, =CHH), 3.42 (*t*, 2H, *J* = 6.75, $BrCH_2$), 2.06 (*q*, 2H, *J* = 7.5, H_2CC=), 1.87 (*quintet*, 2H, *J* = 7.5, H_2CCH_2Br), 1.46-1.37 (*m*, 4H, CH_2), 1.29 (*br.s,* 12H, CH_2).

^{13}C-NMR (126 MHz, $CDCl_3$): δ 139.21 (=CH), 114.06 (=CH_2), 33.93 (H_2CBr), 33.77 (H_2CC=), 32.84 (H_2CCH_2Br), 29.51 (CH_2), 29.48 (CH_2), 29.44 (CH_2), 29.40 (CH_2), 29.10 (CH_2), 28.92 (CH_2), 28.74 (CH_2), 28.17 (CH_2).

^{1}H and ^{13}C NMR spectra are in good agreement with those reported (Nguyen, *et al*., 2006).

Trimethylpentadec-14-en-1-ynysilane

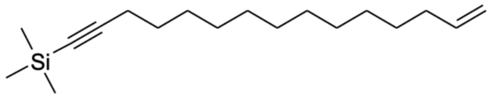

A solution of *n*-BuLi (5.1 ml, 12.7 mmol, Sigma-Aldrich, 2.5M sln. in hexane) was added dropwise to a cooled (-75°C) solution of trimethylsilylacetylene (1.13 g, 11.5 mmol, Sigma-Aldrich) in a mixture THF-HMPTA (25 ml, 10:15, v/v) over 5 min and allowed to heat to -15°C for another 2 h. The clear dark-apricot solution was cooled to -65°C, 13-bromotridec-1-ene (2.09 g, 8.0 mmol, previous step) in THF (2 ml) was added over 5 min, the reaction mixture was allowed to warm to RT over 2 h and stirred for another 2 h. The dark mixture was treated with ammonium chloride water solution (40 ml), the organic layer was separated, the water layer was extracted with diethyl ether (3 x 30 ml), combined extracts were washed with H_2O (4 x 30 ml), brine, dried (Na_2SO_4) and concentrated. The residue was dissolved in petroleum ether, filtered through a SiO_2 layer (3 cm), eluted with petroleum ether (50 ml), concentrated and after chromatography over SiO_2 (200 ml, eluted with petroleum ether) the TMS-C_{15}-alkyl chain was isolated in 1.80 g (81%) as a colourless liquid.

TLC (petroleum ether): R_f 0.5. Liquid.

^{1}H-NMR (500 MHz, $CDCl_3$): 5.86-5.78 (*m*, 1H, HC=), 5.01 (*d*, 1H, *J* = 17.1, =C<u>H</u>H), 4.94 (*d*, 1H, *J* = 10.1, =CH<u>H</u>), 2.23 (*t*, 2H, *J* = 7.2, H_2CC≡), 2.06 (*q*, 2H, *J* = 7.1, H_2CC=), 1.56-1.50 (*m*, 2H, CH_2), 1.42-1.36 (*m*, 2H, CH_2), 1.29 (*br.s*, 14H, 7 x CH_2), 0.16 (*s*, 9H, $(H_3C)_3$Si.

^{13}C-NMR (126 MHz, $CDCl_3$): 139.22 (=CH), 114.04 (=CH_2), 107.77 (C<u>C</u>≡), 84.23 (SiC≡), 33.77 (H_2<u>C</u>C=), 29.55 (CH_2), 29.54 (CH_2), 29.47 (CH_2), 29.45 (CH_2), 29.12 (CH_2), 29.05 (CH_2), 28.93 (CH_2), 28.77 (CH_2), 28.63 (CH_2), 19.83 (H_2<u>C</u>C≡), 0.16 $(H_3C)_3$Si.

MS (ESI): m/z (positive mode) 297.3 $[M+H+H_2O]^+$, 319.2 $[M+Na+H_2O]^+$, 593.5 $[2M+H+2H_2O]^+$.

^{1}H and ^{13}C NMR spectra are in agreement with those reported for the closest known C_{13}-homologue (Predeus *et al,.* 2013).

(2*S*,3*R*,4*E*)-2-(*tert*-Butoxycarbonyl)amino-1-(*tert*-butyldimethylsilyloxy)-18-trimethylsilyloctadec-4-en-17-yn-3-ol (S5)

TMS-C_{15}-olefin chain was cross-metathesised with the head group prepared from Boc-L-serine (Yamamoto *et al*., 2006; Mina *et al*., 2011) in CH_2Cl_2 at 83°C for 40 h with the Grubbs catalyst (2nd gen) on analogy to our previous work (Kol *et al*., 2025). After chromatography over SiO_2, fully protected click sphingosine **S5** (20%) and several by-products were isolated: self-dimer of the head group (10%), self-dimer of the TMS-C_{15}-olefin chain (36%) and a saturated ketone (36%), the isomerization product of the head group. ^{1}H and ^{13}C NMR spectra and TLC data of all isolated compounds matched well with those obtained by us earlier (Kol *et al*., 2025).

TLC (CH_2Cl_2-AcOEt, 100:1, v/v): R_f 0.49. Oil.

(2*S*,3*R*,4*E*)-2-(*tert*-Butoxycarbonyl)aminooctadec-4-en-17-yn-1,3-diol (S6)

Simultaneous removal of both Si-containing protecting groups (TMS and TBDMS) from the sphingosine **S5** was accomplished with TBAF in THF at 40°C for 6 h. After chromatography, Boc-click-sphingosine **S6** was isolated in 81% yield. ^{1}H and ^{13}C NMR spectra, as well as TLC data were consistent with those obtained by us earlier (Kol *et al.*, 2025).

TLC (CH_2Cl_2-AcOEt, 1:1, v/v): R_f 0.61. Oil.

(2*S*,3*R*,4*E*)-2-Amino-4-octadecen-17-yne-1,3-diol (cSph)

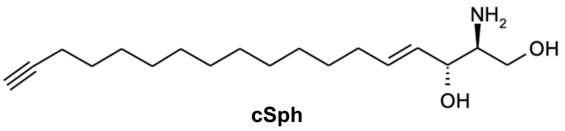

cSph

The removal of the Boc-protecting group from sphingosine **S6** was carried out under conditions of acid catalysis and after chromatography the clickable sphingosine **cSph** was isolated in 64% yield with NMR spectra corresponded to our previous data (Kol et al., 2025).

TLC (AcOEt-MeOH-NH_3/MeOH, 70:10:2, v/v/v): R_f 0.35. White powder.

1.2.2 Synthesis of cgMito-cCer$_6$

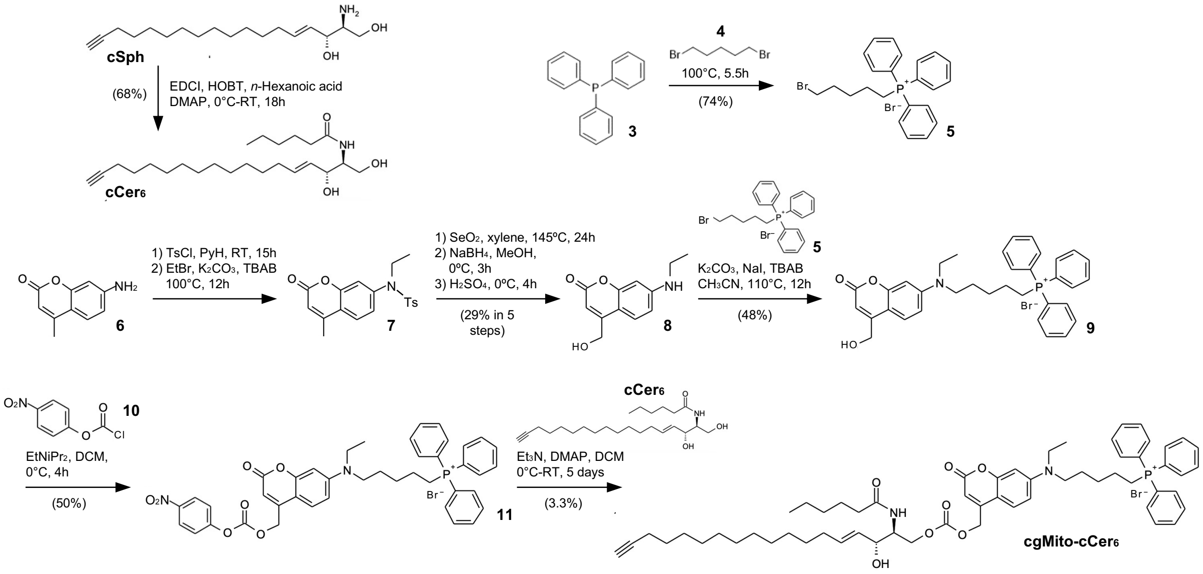

Scheme 2. Scheme for the synthesis of **cgMito-cCer$_6$**.

The synthesis of the caged ceramide **cgMito-cCer$_6$** was accomplished in 22 steps (Scheme 2) and involved the separate preparation of click-ceramide **cCer$_6$**, which included the caproic acid ester chain, and a mitochondria-targetable photocaged unit **cgMito** based on a coumarin dye with a mitochondria-targetable head as phosphonium salt. In the final stage, both blocks were connected with a photo-cleavable carbonate spacer.

_N_-[(1_S_,2_R_,3_E_)-(2-hydroxy-1-hydroxymethylheptadec-3-en-16-ynyl)-hexanamide (cCer$_6$)

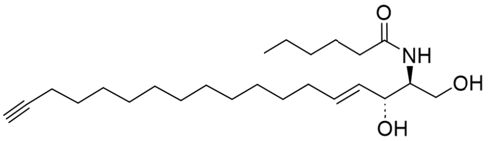

cCer6

A solution of EDCI (13 mg, 0.084 mmol, TCI) in CH_2Cl_2 (0.2 ml) was added dropwise to a pre-cooled (ice-bath) solution of click-sphingosine (cSph) (20.8 mg, 0.07 mmol), caproic acid (9.4 mg, 0.081 mmol, Sigma-Aldrich), DMAP (12.8 mg, 0.11 mmol) and HOBT (11.3 mg, 0.084 mmol) in CH_2Cl_2 (0.5 ml) and the resulting mixture was stirred in a cooling bath until the sphingosine (cSph) is completely consumed (TLC analysis). The clear yellowish reaction mixture was acidified with 1M HCl (0.5 ml), organic phase was separated and the aqueous phase was extracted with CH_2Cl_2 (2 x 2 ml). The combined organic phases were washed with water, 10% aq. $NaHCO_3$ and brine, then dried over anhydrous Na_2SO_4 and concentrated under reduced pressure (1 mm). The resulting light apricot oil after chromatography on TLC (eluted with a mixture CH_2Cl_2-MeOH 90:6 v/v) resulted in isolation of desired product and undesired diacylated product (in the order of their elution): (2S, 3R, 4E)-(2-hexanoylamino-3-hydroxyoctadec-4-en-17-ynyl) hexanoate (**S7**, diacylated product, 5.7 mg, 28%), N-[(1S, 2R, 3E)-(2-hydroxy-1-hydroxymethylheptadec-3-en-16-ynyl)-hexanamide (**cCer$_6$**, 18.9 mg, 68%).

TLC (AcOEt): R_f 0.35. White solid.

^{1}H-NMR (500 MHz, $CDCl_3$): δ 6.23 (d, J = 7.5 Hz, 1H, NH), 5.79 (dtd, J = 15.1, 6.8, 1.2 Hz, 1H, HC=), 5.54 (td, J = 15.1, 6.8, 1.2 Hz, 1H, =CHC-O), 4.35 – 4.29 (m, 1H, HC-N), 3.98 – 3.88 (m, 2H, H_2C-O), 3.71 (dd, J = 11.2, 3.3 Hz, 1H, HCO), 2.23 (t, J = 11.2, 2H, H_2CC=O), 2.18 (td, J = 7.1, 2.7 Hz, 2H, H_2CC≡), 2.06 (q, J = 7.0 Hz, 2H, H_2CC=), 1.93 (t, J = 2.7 Hz, 1H, HC≡), 1.65 (quint, J = 7.4 Hz, 2H, CH_2), 1.52 (quint, J = 7.1 Hz, 2H, CH_2), 1.43 – 1.22 (m, 23H, CH_2), 0.93 – 0.87 (t, J = 10 Hz, 3H);

^{13}C-NMR (126 MHz, $CDCl_3$): δ 174.03 (NC=O), 134.43 (HC=), 129.05 (HC=), 84.96 (-C≡), 74.83 (HC-OH), 68.17 (HC≡), 62.69 (H_2C-O), 54.76 (HC-N), 36.96 ($H_2\underline{C}$-CON), 32.42 ($H_2\underline{C}$-C=), 31.59 (H_2C), 29.73 (H_2C), 29.63 (H_2C), 29.60 (H_2C), 29.35 (H_2C), 29.26 (H_2C), 28.91 (H_2C), 28.67 (H_2C), 25.58 (H_2C), 22.54 (H_2C), 18.56 ($H_2\underline{C}$-C≡), 14.06 (H_3C).

MS (ESI) m/z (positive mode): 376 $[M+H – H_2O]^+$, 394 $[M+H]^+$, 416 $[M+Na]^+$.

Undesired diacylated side product of cCer$_6$ (S7)

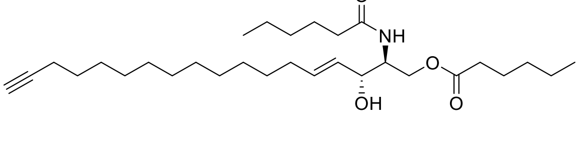

TLC (AcOEt): R_f 0.88. White powder.

^{1}H-NMR (500 MHz, $CDCl_3$): 5.83 (*d*, 1H, *J* = 7.95, 1H, NH), 5.76 (*dt*, 1H, *J* = 15.45, 6.7, HC=), 5.49 (*dd*, 1H, *J* = 15.45, 6.5, =CHC-O), 4.34 (*dd*, 1H, *J* = 11.45, 6.75, HC-N), 4.28-4.23 (*m*, 1H, HC-O),

4.20-4.15 (*m*, 2H, H_2CO), 2.33 (*t*, 2H, *J* = 7.6, H_2CC=O), 2.22-2.18 (*m*, 4H, H_2CC=O, H_2CC≡), 2.05 (*br.q*, 2H, *J* = 7.15, H_2CC=), 1.94 (*t*, 1H, *J* = 2.5, HC≡), 1.64 (*quint*, 4H, *J* = 7.25, CH_2), 1.54 (*quint*, 2H, *J* = 7.25, CH_2), 1.42-1.28 (*m*, 24H, CH_2), 0.92 (*t*, 6H, *J* = 6.75, 2 x H_3C)

^{13}C-NMR (126 MHz, $CDCl_3$): 174.07 (OC=O), 173.73 (NC=O), 134.56 (HC=); 132.01 (HC=), 84.77 (-C≡), 73.38 (HC-OH), 67.98 (HC≡), 62.74 (H_2C-O), 53.48 (HC-N), 36.74 ($H_2\underline{C}$-CON), 34.16 ($H_2\underline{C}$-COO), 32.26 ($H_2\underline{C}$-C=), 31.36 (H_2C), 31.27 (H_2C), 29.56 (H_2C), 29.53 (H_2C), 29.45 (H_2C), 29.43 (H_2C), 29.19 (H_2C), 29.09 (H_2C), 29.07 (H_2C), 28.74 (H_2C), 28.49 (H_2C), 25.35 (H_2C), 24.55 (H_2C), 22.35 (H_2C), 22.26 (H_2C), 18.38 ($H_2\underline{C}$-C≡), 13.86 (H_3C), 13.83 (H_3C).

MS (ESI): m/z (positive mode): 278 $[M+H – (2 x C_6) - H_2O]^+$, 376 $[M+H – (C_6) - H_2O]^+$, 394 $[M+H – (C_6)]^+$, 474 $[M+H - H_2O]^+$, 492 $[M+H]^+$, 514 $[M+Na]^+$, 885 $[2M+H – (C_6)]$, 983 $[2M+H]^+$.

(5-Bromopentyl)-triphenylphosphonium bromide (**5**)

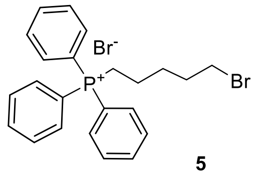

5

(5-Bromopentyl)-triphenylphosphonium bromide (**5**) was synthesized as previously described (Deanel *et al.*, 2014) and the NMR spectra were recorded in CD_2Cl_2 without any purification. 1,5-dibromopentane (41 g, 179.2 mmol) and triphenylphosphine (10 g, 35.8 mmol) were heated together at 90°C for 5.5 hrs. Toluene (30 mL) was then added and the solution was decanted. The cloudy oil was then washed with toluene (2 × 50 mL). Diethyl ether (100 mL) was added to the resulting oil, and the mixture was sonicated for 1 hr, then the creamy precipitate was filtered, washed with diethyl ether (2 x 50 mL), dried in vacuum to give (5-bromopentyl)triphenylphosphonium bromide **5** (13.05 g, 26.5 mmol, 74%) as off-white powder.

TLC (CH_2Cl_2-MeOH, 10:1, v/v): R_f 0.6 (Hygroscopic off-white powder).

^{1}H-NMR (500 MHz, CD_2Cl_2): δ 7.86 – 7.78 (m, 9H, Ar-H), 7.74 – 7.69 (m, 6H, Ar-H), 3.73 – 3.65 (m, 2H, P-CH_2), 3.39 (t, J = 6.6 Hz, 2H, Br-CH_2), 1.94 – 1.86 (m, 2H, CH_2), 1.82 – 1.65 (m, 4H, CH_2).

^{13}C-NMR (126 MHz, CD_2Cl_2): δ 135.52 (Ar-CH), 135.50 (Ar-CH), 134.15 (Ar-CH), 134.07 (Ar-CH), 130.88 (Ar-CH), 130.78 (Ar-CH), 118.94 (Ar-C (quat.), 118.25 (Ar-C (quat.), 33.88 (CH_2), 32.37 (CH_2), 29.38 (CH_2), 29.25 (CH_2), 23.52 (CH_2), 23.11 (CH_2), 22.23 (CH_2), 22.20 (CH_2).

^{31}P-NMR (101 MHz, CD_2Cl_2): δ 24.07.

Ethylamino-4-hydroxymethyl-coumarin (8)

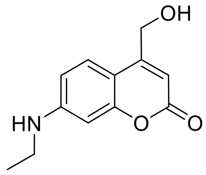

8

7-Ethylamino-4-hydroxymethyl-coumarin (**8**) was synthesized from 7-Amino-4-methyl-coumarin (ABCR) in 5 steps with an overall yield of 29% as previously described (Lin *et al.,* 2012; Vuilleumier *et al.,* 2019).

TLC (1:1, CyH:AcOEt, v/v): R_f 0.3.

^{1}H NMR (500 MHz, MeOD): δ 7.39 – 7.35 (d, J = 8.8 Hz, 1H, Ar-CH), 6.62 – 6.57 (dd, J = 8.8, 2.3 Hz, 1H, Ar-CH), 6.46 – 6.41 (d, J = 2.3 Hz, 1H, Ar-CH), 6.25 – 6.15 (s, 1H, =CH), 4.77 – 4.77 (d, J = 1.4 Hz, 2H, O-CH_2), 3.22 – 3.15 (q, J = 7.2 Hz, 2H, N-CH_2), 1.28 – 1.23 (t, J = 7.2 Hz, 3H (CH_3)).

^{13}C NMR (126 MHz, MeOD): δ 165.1 (Ar-CH), 158.9 (Ar-CH), 157.5 (Ar-CH), 154.3 (Ar-CH), 125.6 (Ar-CH), 112.0 (Ar-C-quat.), 108.2 (Ar-C-quat.), 104.9 (Ar-C-quat.), 97.9 (=C), 60.9 (O-CH_2), 38.6 (N-CH_2), 14.4 (CH_3).

{5-[Ethyl-(4-hydroxymethyl-2-oxo-2H-chromen-7-yl)-amino]-pentyl}-triphenyl-phosphonium bromide (cgMito)

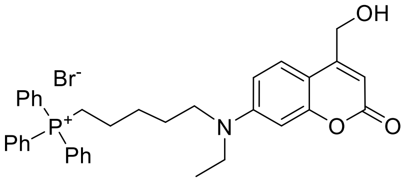

cgMito

Fine powders of K_2CO_3 (52 mg, 0.377 mmol), NaI (38 mg, 0.25 mmol) and TBAB (8 mg, 0.03 mmol) were added sequentially to a solution of ethyl coumarin **8** (54.8 mg, 0.25 mmol [E2]) and phosphonium salt **5** (160 mg, 0.325 mmol) in acetonitrile (2 ml) and this mixture was heated for 10 h at 100 °C (bath temperature). The phosphonium salt **5** (4 x 110 mg, 4 x 0.22 mmol) in acetonitrile (0.5 ml) was added to the reaction mixture 4 more times, followed by heating for 4 x 10 h.

The reaction mixture was cooled to room temperature and filtered through a SiO_2 layer (1 cm) from inorganic salts, washed with a mixture of MeCN-MeOH 10:1 (10 ml) and concentrated in vacuum. Chromatography of the apricot residue on silica gel (50 ml, eluted with a gradient mixture CH_2Cl_2-AcOEt-MeOH from 85:15:0 to 9:0:1 v/v/v) gave the following identified substances (in the order of their elution): starting ethyl coumarin **8** (5.4 mg, 10%), pent-4-enyltriphenylphosphonium bromide (81 mg, 15%), coumarin phosphonium salt **cgMito** (83 mg, 52%).

TLC (CH_2Cl_2-AcOEt-MeOH, 2:2:1, v/v/v): R_f 0.56.

^{1}H-NMR (500 MHz, $CDCl_3$): δ 7.88-7.82 (m, 15H), 7.81-7.78 (m, 3H), 7.73-7.69 (m, 6H), 7.32 (d, J = 9.0, 1H), 6.48 (*dd*, *J* = 9.0, 2.0, 1H), 6.30 (*d*, *J* = 2.0, 1H), 6.21 (*s*, 1H, HC=), 4.81 (*s*, 2H, H_2CO),

4.4-4.3 (*br.s*, 1H, HO), 3,89-3.79 (*m*, 2H, H_2CP), 3.33 (*q*, 2H, J = 7.0, H_2CN), 3.21-3.19 (*m*, 2H, H_2CN), 1.69-1.59 (*m*, 6H, $(CH_2)_3$), 1.10 (*t*, 3H, *J* = 7.0, H_3C).

^{13}C-NMR (126 MHz, $CDCl_3$): δ 162.61 (OC=O), 156.1(>C=), 155.87(>C=), 150.45 (NC=), 135.06 (*d*, $^4J_{CP}$ = 2.8, *p*-CH_{Ar}), 133.62 (*d*, $^3J_{CP}$ = 9.8, *m*-CH_{Ar}), 130.53 (*d*, $^2J_{CP}$ = 12.6, *o*-CH_{Ar}), 124.72 (HC=), 118.25 (*d*, $^1J_{CP}$ = 85.6, P-C_{Ar}), 108.76 (HC=), 106.66 (>C=), 105.33 (HC=), 97.41 (HC=), 60.04 (H_2COH), 49.94 (H_2CN), 45.11 (H_2CN), 27.77 (*d*, $^2J_{CP}$ = 16.3, H_2C), 26.89 (H_2C), 22.68 (*d*, $^1J_{CP}$ = 50.3, H_2CP), 22.64 (*d*, $^3J_{CP}$ = 4.3, H_2C), 12.10 (H_3C).

^{31}P-NMR (101 MHz, $CDCl_3$): δ 24.2.

MS (ESI) m/z (positive mode): 242 [M+Na – Ph_3P-$(CH_2)_3CH=CH_2$]$^+$, 550 [M]$^+$.

Undesired eliminated product pent-4-enyltriphenylphosphonium bromide

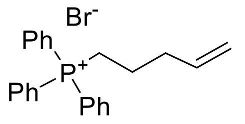

TLC (CH_2Cl_2-AcOEt-MeOH, 4:4:1, v/v/v): R_f 0.43.

^{1}H-NMR (500 MHz, $CDCl_3$): δ 7.81-7.78 (*m*, 9H, CH_{Ar}), 7.74-7.67 (*m*, 6H, CH_{Ar}), 5.76-5.68 (*m*, 1H, =CH), 5.07 (*dd*, 1H, *J* = 17.0, 1.5, =C<u>H</u>H), 5.03 (*dd*, 1H, *J* = 10.0, 1.5, =CH<u>H</u>), 3.73-3.65 (*m*, 2H, PCH_2), 2.45-2.41 (*br.q*, 2H, *J* = 6.0, CH_2), 1.8-1.7 (*m*, 2H, CH_2).

^{31}P-NMR (101 MHz, $CDCl_3$): δ 24.3.

^{1}H-NMR spectra are in a good agreement with those reported (Chu *et al.,* 2009).

(5-{Ethyl-[4-(4-nitrophenoxycarbonyloxymethyl)-2-oxo-2H-chromen-7-yl]-amino}-pentyl)-triphenyl-phosphonium bromide (11)

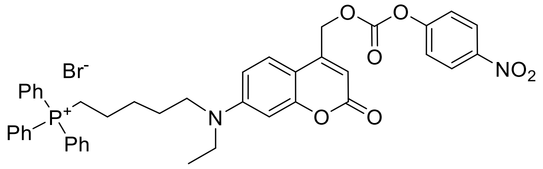

11

A solution of 4-nitrophenyl chloroformate **10** (30 mg, 0.15 mmol, Alfa Aesar) in CH_2Cl_2 (0.3 ml) was added drop wise to a pre-cooled (ice bath) solution of coumarin phosphonium salt **9** and diisopropylethylamine (19 mg, 0.15 mmol, Sigma-Aldrich) in CH_2Cl_2 (0.3 ml) during 1 min with protection from light, stirred 1 h, cooling bath was removed and the reaction mixture was stirred at room temperature overnight. The reaction mixture was washed sequentially with HCl_{aq} (0.5%) and H_2O, dried (Na_2SO_4), concentrated and the yellow residue was chromatographed on silica gel (1ml, eluted with a gradient mixture CH_2Cl_2-AcOEt-MeOH from 1:0:0 to 40:20:10). The yellow zone was collected and concentrated to obtain 4-nitrophenoxy carbonyl phosphonium salt **11** (29.4 mg, 50%, yellow film), taken as such to the next step.

TLC (CH_2Cl_2-AcOEt-MeOH, 40:20:15, v/v/v): R_f 0.5.

^{1}**H-NMR** (500 MHz, $CDCl_3$): δ 8.32-8.29 (m, 2H), 7.88-7.84 (m, 6H), 7.82-7.80 (m, 3H), 7.73-7.69 (m, 6H), 7.46-7.42 (m, 2H), 7.32 (d, 1H, *J* = 9.0), 6.66 (*dd*, 1H, *J* = 9.0, 2.0), 6.42 (*d*, 1H, *J* = 2.0), 6.21 (*s*, 1H, HC=), 5.41 (*s*, 2H, H_2CO), 4,04-3.92 (*m*, 2H, H_2CP), 3.44 (*q*, 2H, *J* = 7.0, H_2CN), 3.36-3.33 (*m*, 2H, H_2CN), 1.9-1.6 (*m*, 6H, $(CH_2)_3$), 1.18 (*t*, 3H, *J* = 7.0, H_3C).
^{13}**C-NMR** (126 MHz, $CDCl_3$): δ 161.69 (>C<), 161.59 (OC=O), 156.27(>C=), 155.26(OC=O), 152.10 (=C-O), 150.88 (NC=), 145.65 (CNO_2), 135.10 (*d*, $^4J_{CP}$ = 2.8, *p*-CH_{Ar}), 133.67 (*d*, $^3J_{CP}$ = 9.8, *m*-CH_{Ar}), 130.56 (*d*, $^2J_{CP}$ = 12.6, *o*-CH_{Ar}), 125.36 (HC=), 121.73 (HC_{Ar}), 118.12 (*d*, $^1J_{CP}$ = 85.5, P-C_{Ar}), 109.29 (HC=), 106.43 (>C=), 106.67 (HC=), 97.41 (HC_{Ar}), 65.82 (H_2CO), 50.12 (H_2CN), 45.39 (H_2CN), 27.75 (*d*, $^2J_{CP}$ = 16.3, H_2C), 26.94 (H_2C), 22.78 (*d*, $^1J_{CP}$ = 50.1, H_2CP), 22.72 (*d*, $^3J_{CP}$ = 4.2, H_2C), 12.18 (H_3C).
^{31}**P-NMR** (101 MHz, $CDCl_3$): δ 23.7.
MS (ESI) m/z (positive mode): 550[M - p-$NO_2C_6H_4$-OCO]$^+$, 568[M + H_2O - p-$NO_2C_6H_4$-OCO]$^+$, 715[M]$^+$.

(2S, 3R, 4E)-(5-{Ethyl-[4-(2-hexanoylamino-3-hydroxyoctadec-4-en-17-ynyloxycarbonyl-oxymethyl)-2’-oxo-2’H-chromen-7’-yl]-amino}-pentyl)-triphenyl-phosphonium bromide (cgMito-cCer$_6$)

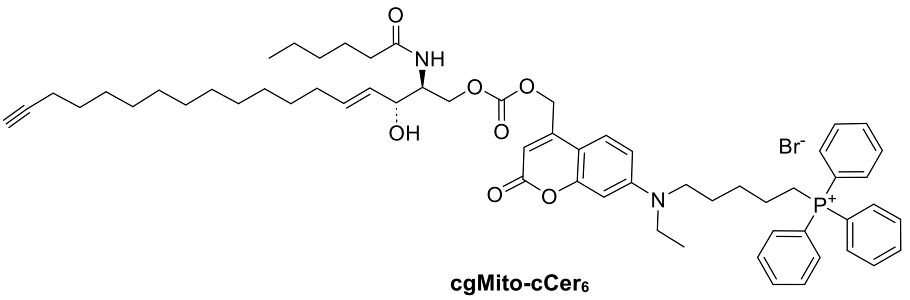

cgMito-cCer$_6$

A solution of DMAP (3.1 mg, 0.025 mmol) and Et_3N (2.6 mg, 0.025 mmol) in CH_2Cl_2 (0.2 ml) was added drop wise to a pre-cooled (ice-bath) solution of **cCer$_6$** (**2**) (9.1 mg, 0.023 mmol) and nitrophenoxycarbonyl phosphonium salt **11** in CH_2Cl_2 (0.3 ml) with protection from light. The resulting reaction mixture was stirred over 5 d at RT, divided into fractions by column chromatography over SiO_2 (eluted with a mixture CH_2Cl_2-AcOEt-MeOH 50:25:10 v/v/v), after which the fraction containing the **cgMito-cCer$_6$** was additionally purified by TLC on SiO_2 (eluted with a mixture CH_2Cl_2-MeOH 60:7) to give caged ceramide **cgMito-cCer$_6$** as a yellow film in a low yield (0.8 mg, 3.3%).
TLC (CH_2Cl_2-Et_2O-MeOH, 40:40:15, v/v/v): R_f 0.52
^{1}**H-NMR (500 MHz, $CDCl_3$):** δ 7.91 – 7.58 (m, 15H, Ar-CH), 6.62 (d, J = 13.9 Hz, 1H, Ar-CH), 6.37 (s, 1H, Ar-CH), 6.09 (d, J = 13.9 Hz, 1H, =CH), 5.76 (dd, J = 15.4, 6.9 Hz, 1H, =CH), 5.50 (d, J = 16.2 Hz, 1H, =CH), 5.32 – 5.13 (m, 2H, O-CH_2), 4.38 – 4.16 (m, 3H, O-CH, P-CH_2), 3.83 – 3.72 (m, 2H, O-CH_2), 3.67 – 3.54 (m, 3H, N-CH, N-CH2), 3.45 – 3.26 (m, 4H, 2 x N-CH_2), 2.31 (t, *J* = 7.5

Hz, 1H, $=CH_2$), 2.21-2.15 (m, 2H, $=CH_2$), 2.07 – 1.99 (m, 2H, $=CH_2$), 1.92 (t, *J* = 2.6 Hz, 1H, HC≡), 1.81 – 1.44 (m, 23H, CH_2), 1.43 – 1.31 (m, 8H, CH_2), 1.17 – 1.14 (m, 3H, CH_3)

^{31}P-NMR (101 MHz, CDCl3): δ 24.23.

MS (ESI) m/z (positive mode): 969.6 $[M]^+$.

1.3 Copies of NMR Spectra

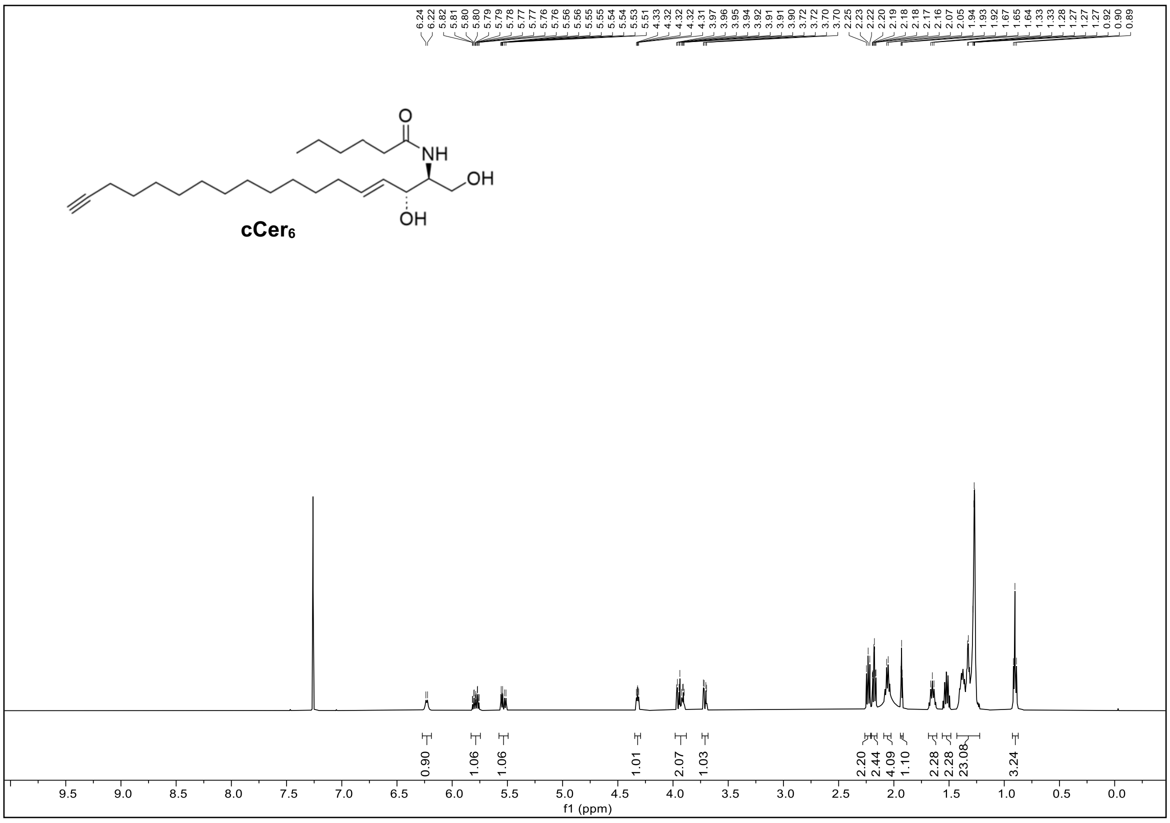

^{1}H-NMR spectrum of **cCer₆**

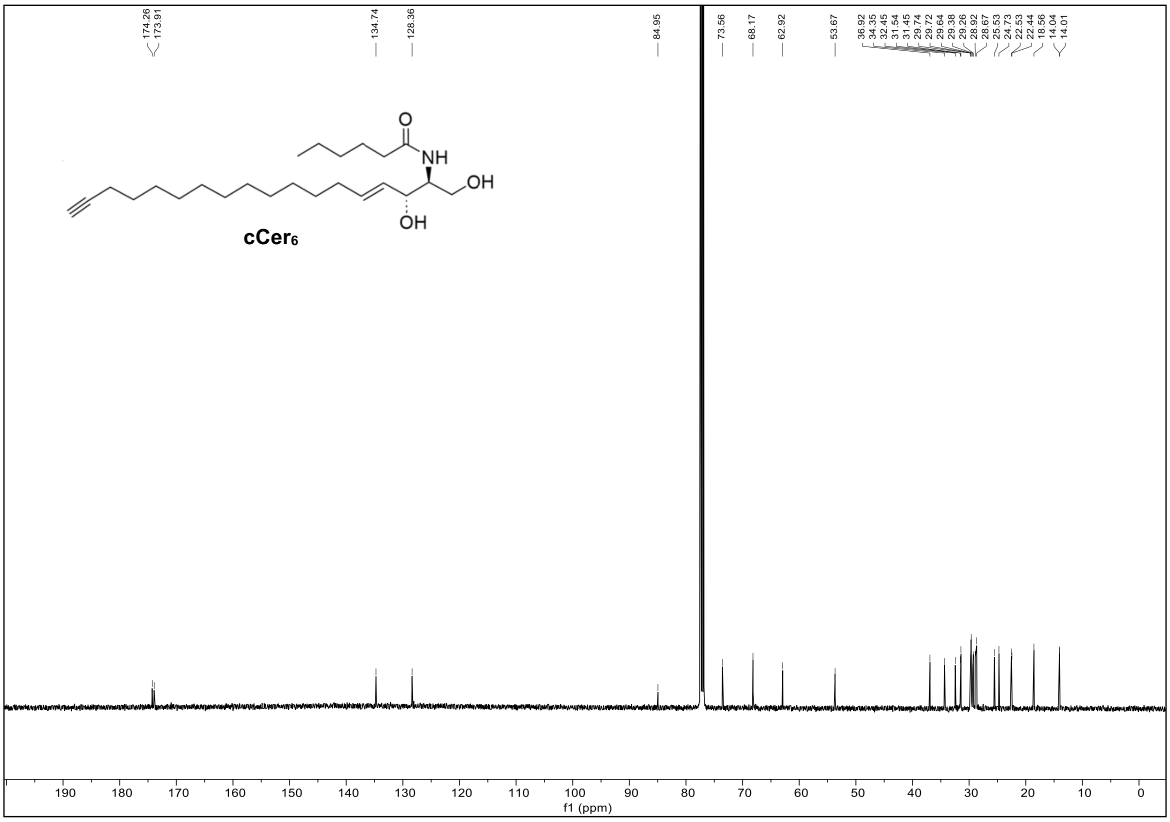

^{13}C-NMR spectrum of **cCer₆**

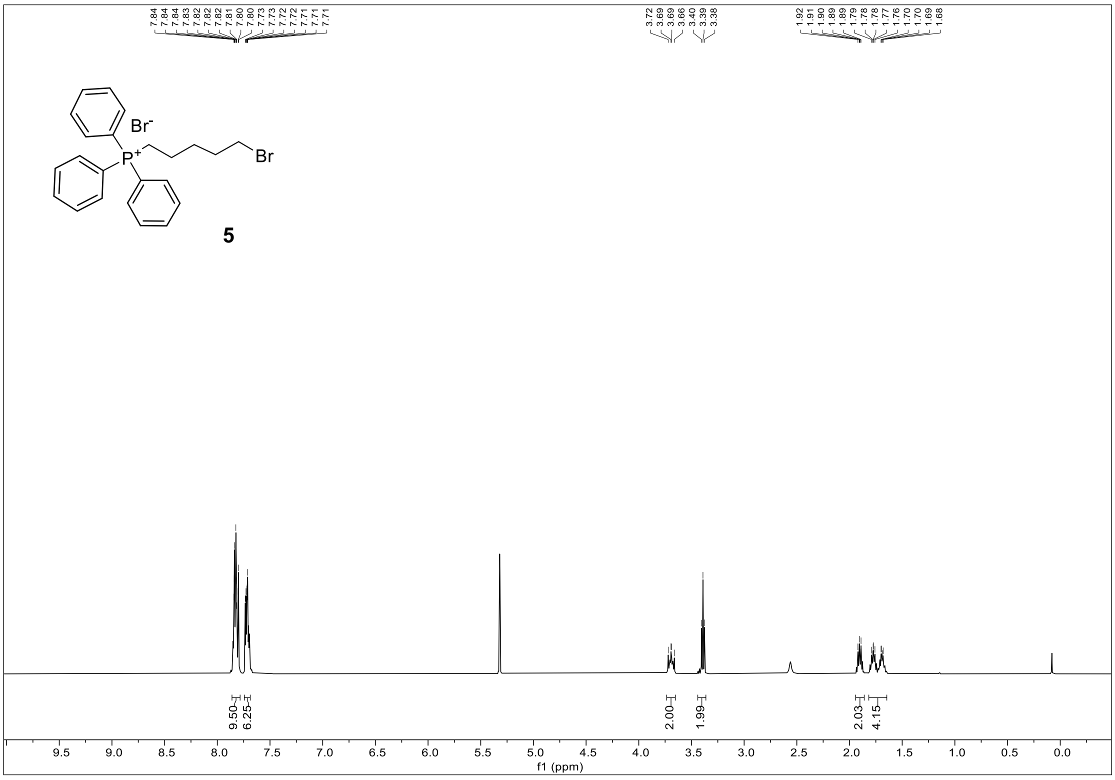

^{1}H-NMR spectrum of phosphonium salt **5**

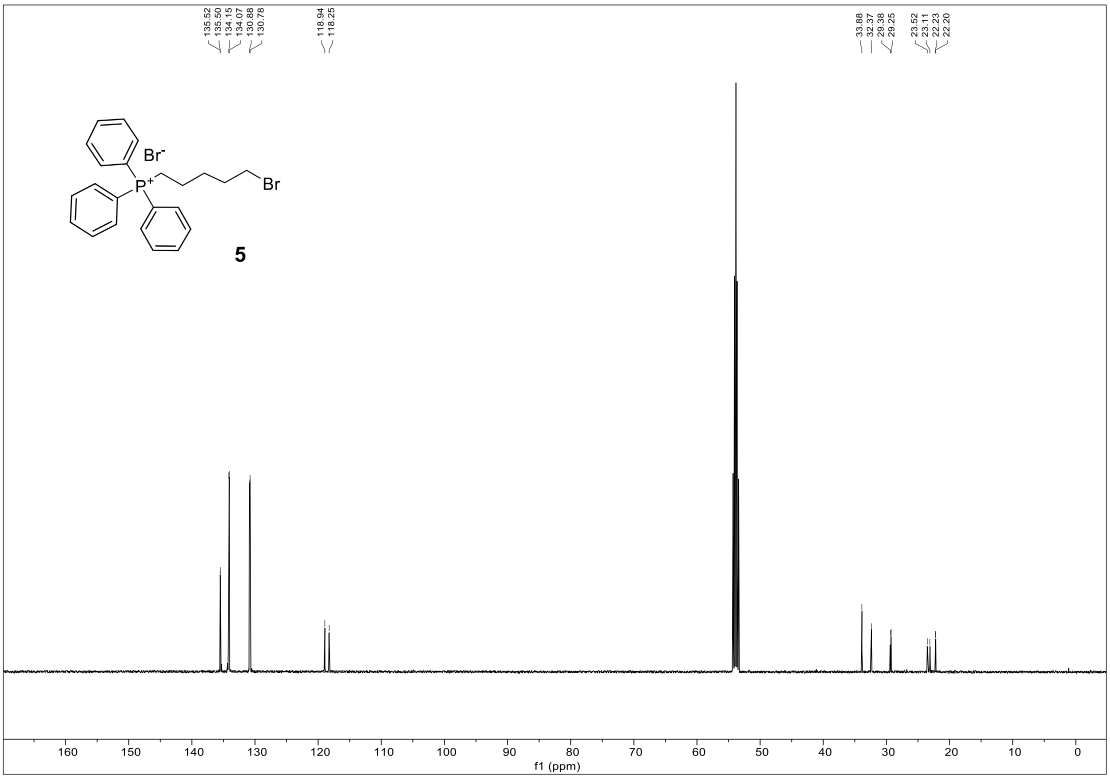

^{13}C-NMR spectrum of phosphonium salt **5**

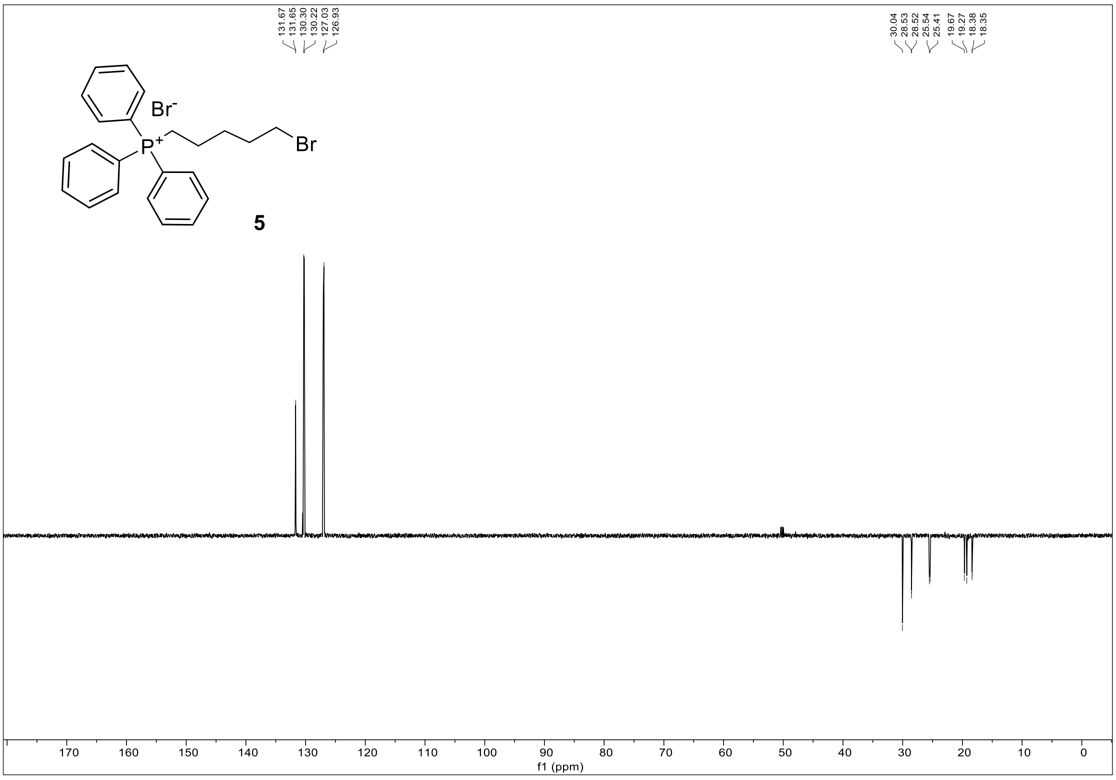

DEPT-NMR spectrum of phosphonium salt **5**

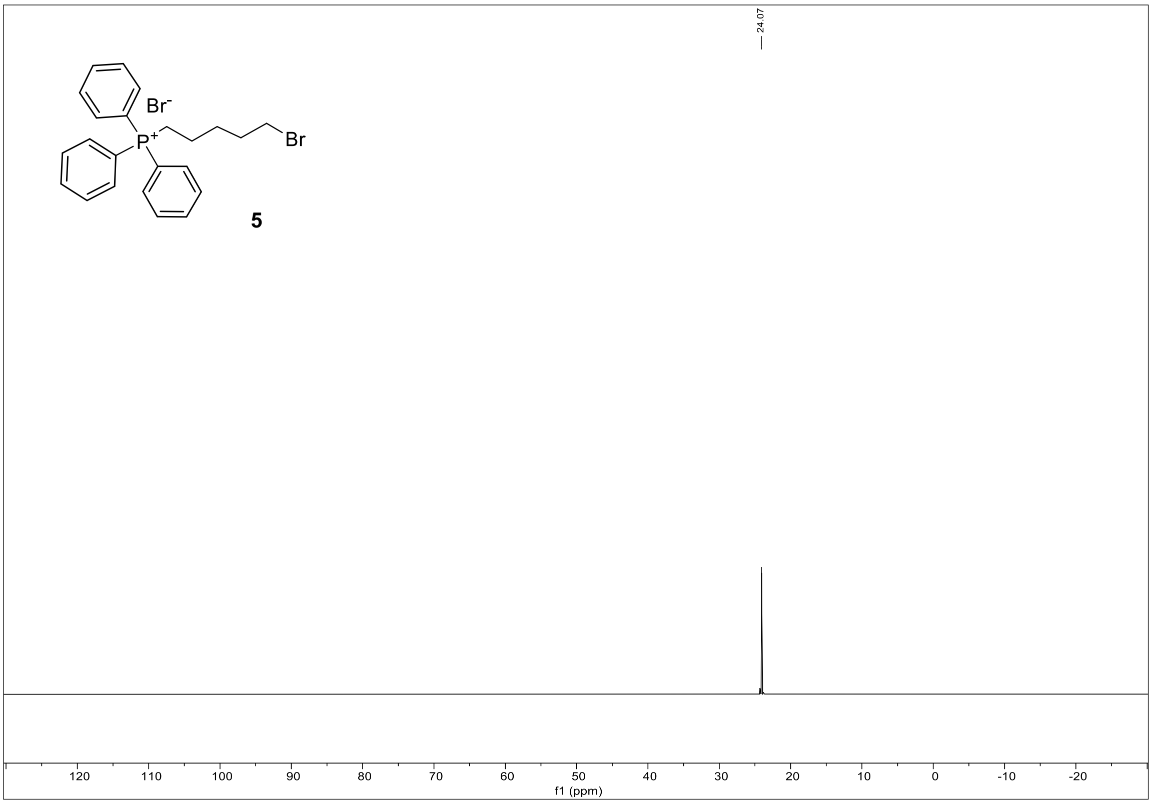

^{31}P-NMR spectrum of phosphonium salt **5**

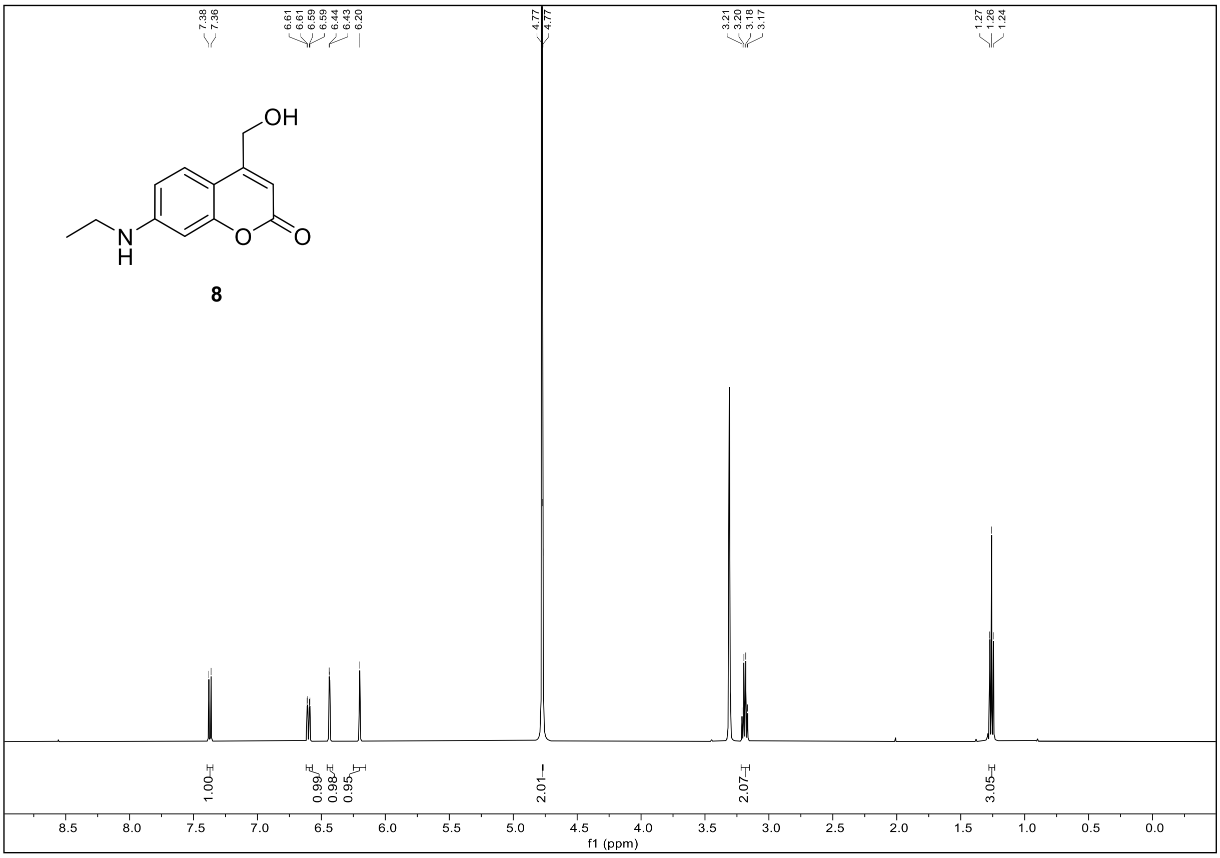

^{1}H-NMR spectrum of **8**

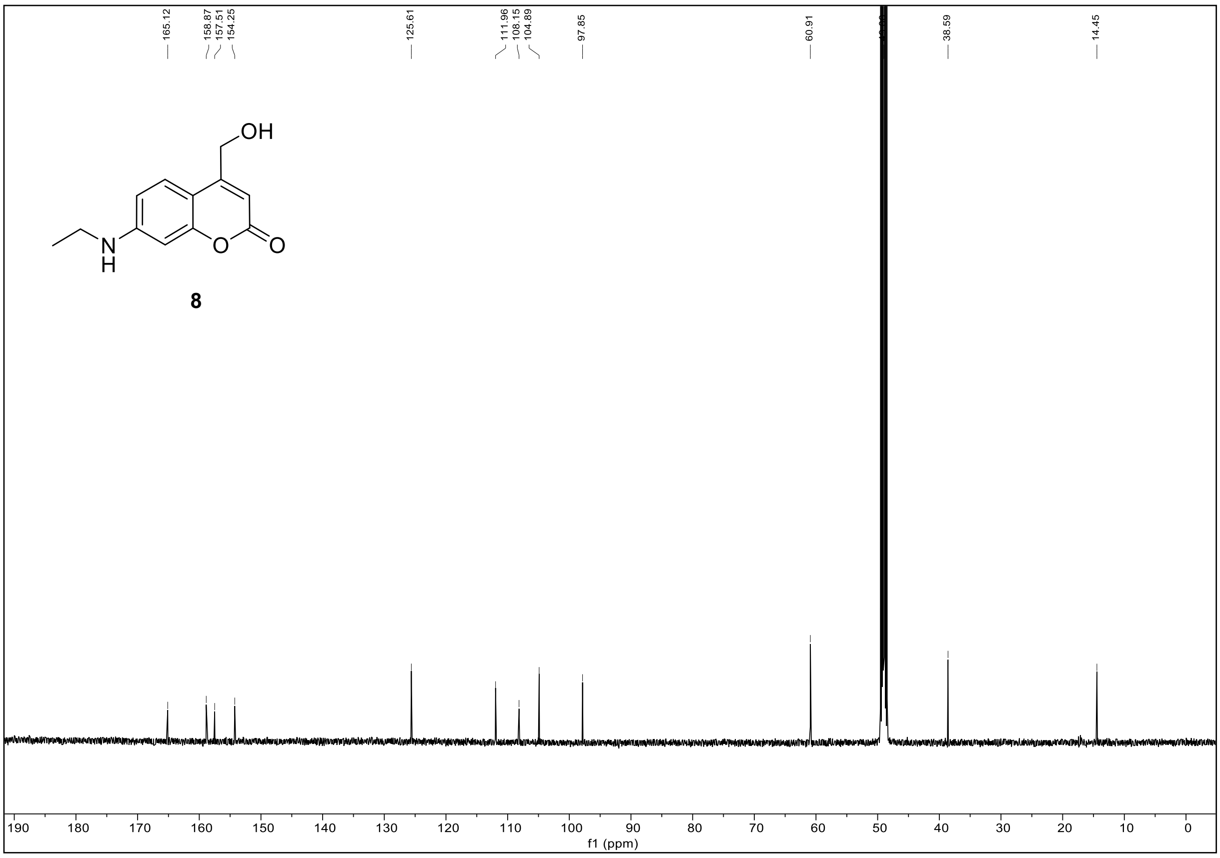

^{13}C-NMR spectrum of **8**

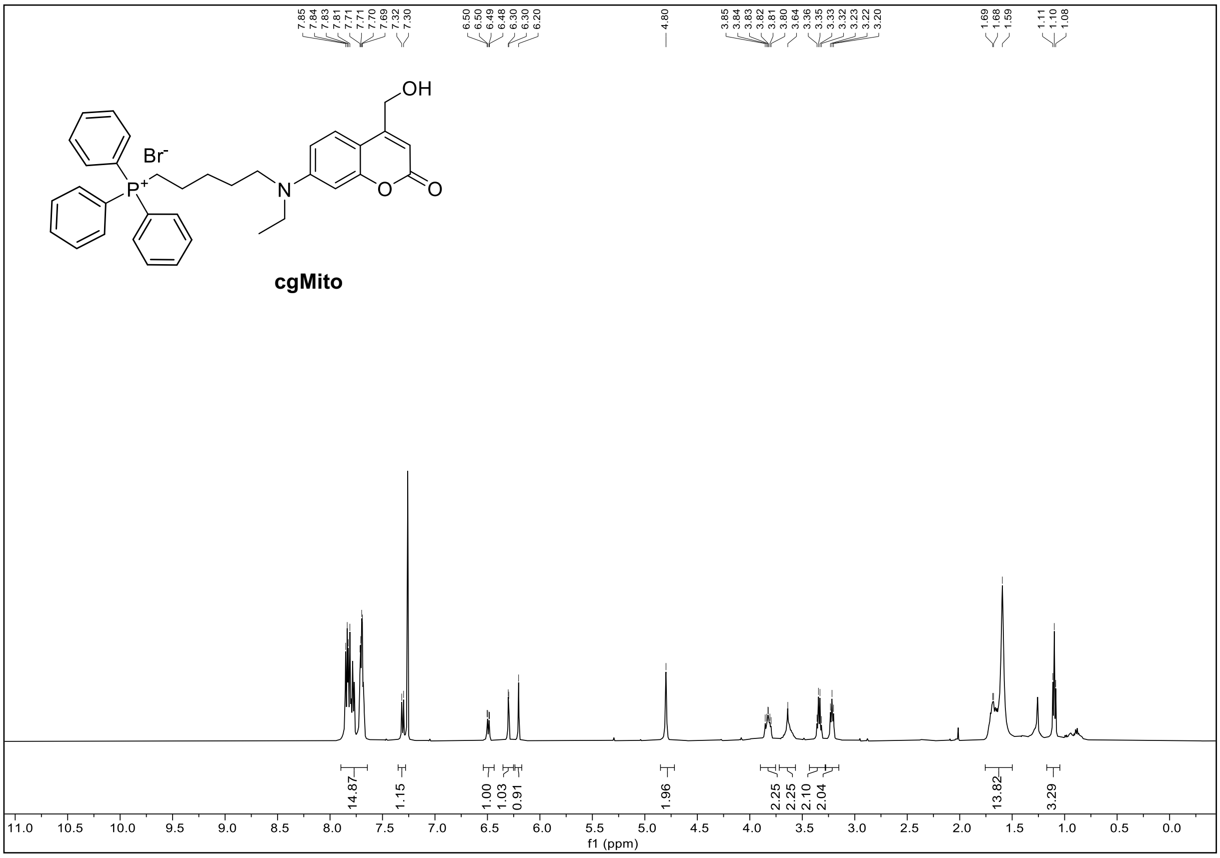

^{1}H-NMR spectrum of **cgMito**

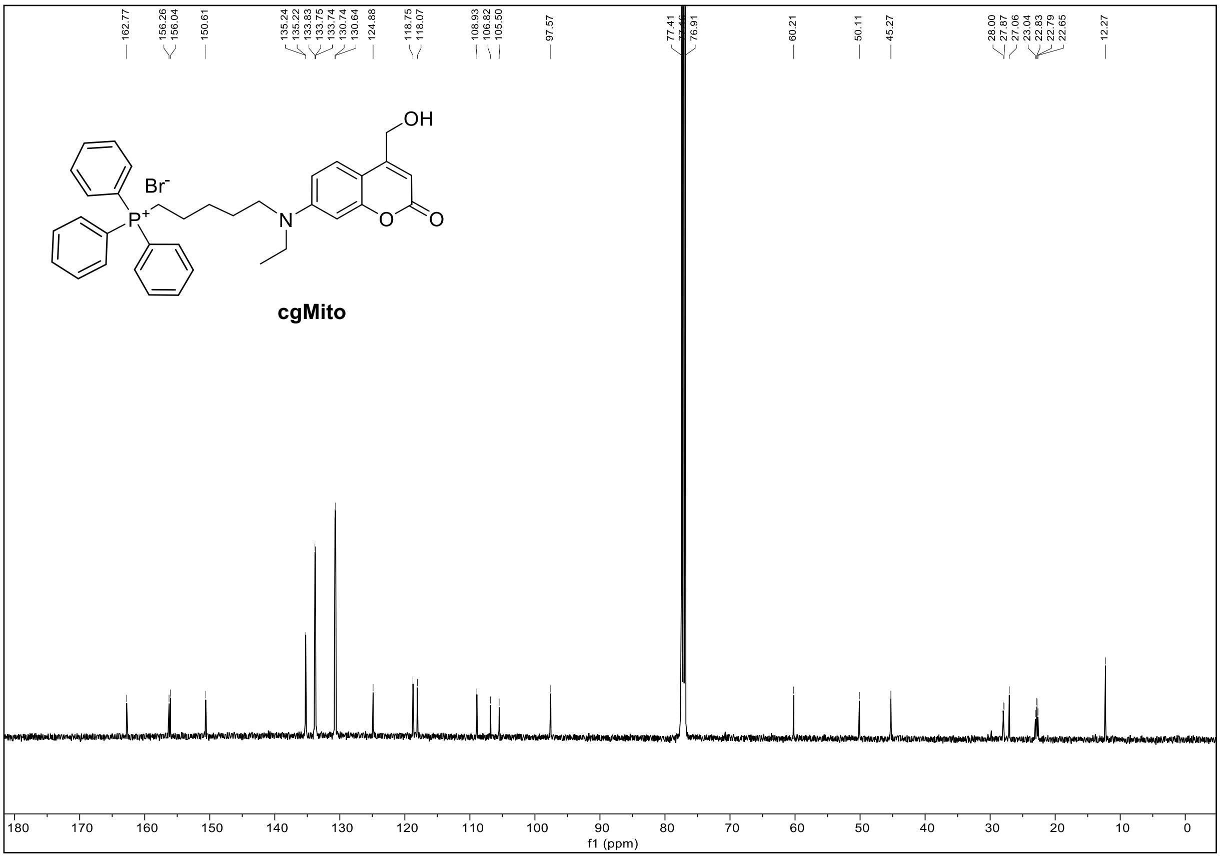

^{13}C-NMR spectrum of **cgMito**

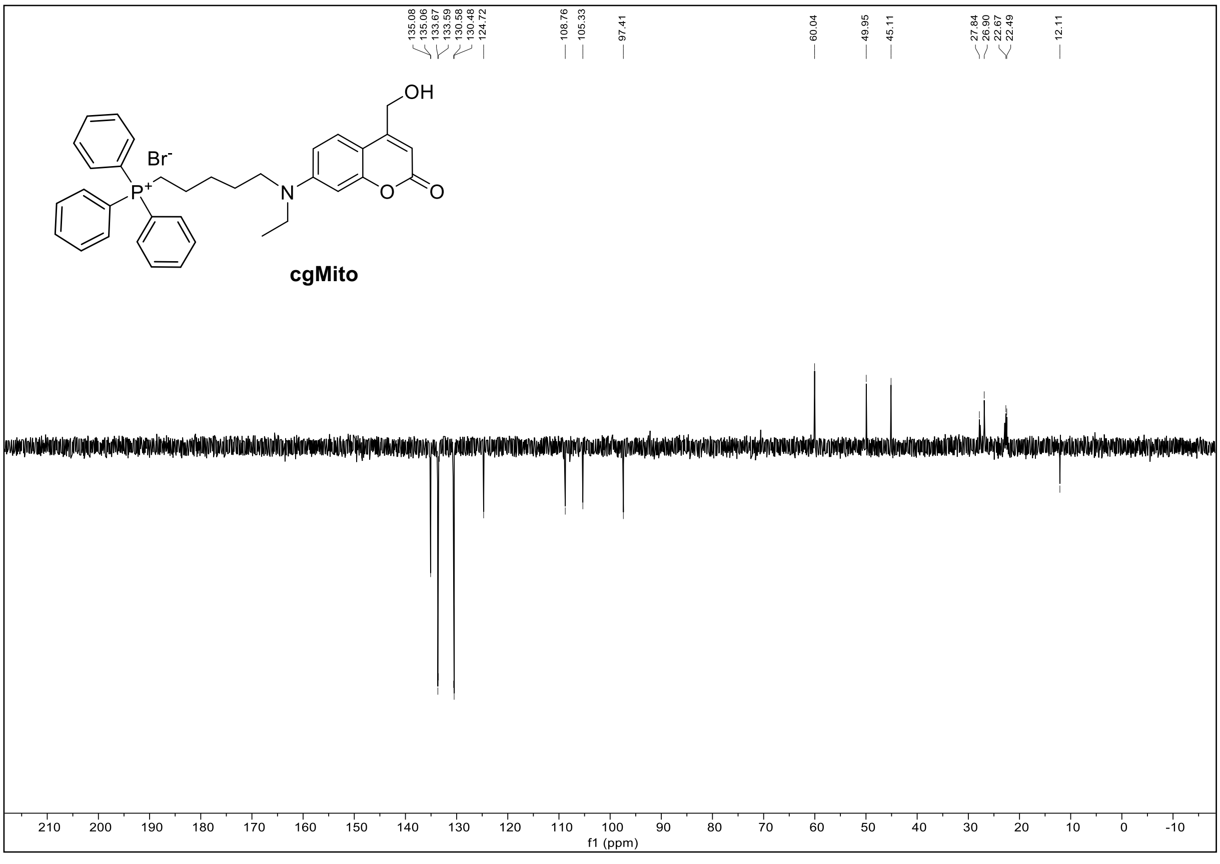

DEPT-NMR spectrum of **cgMito**

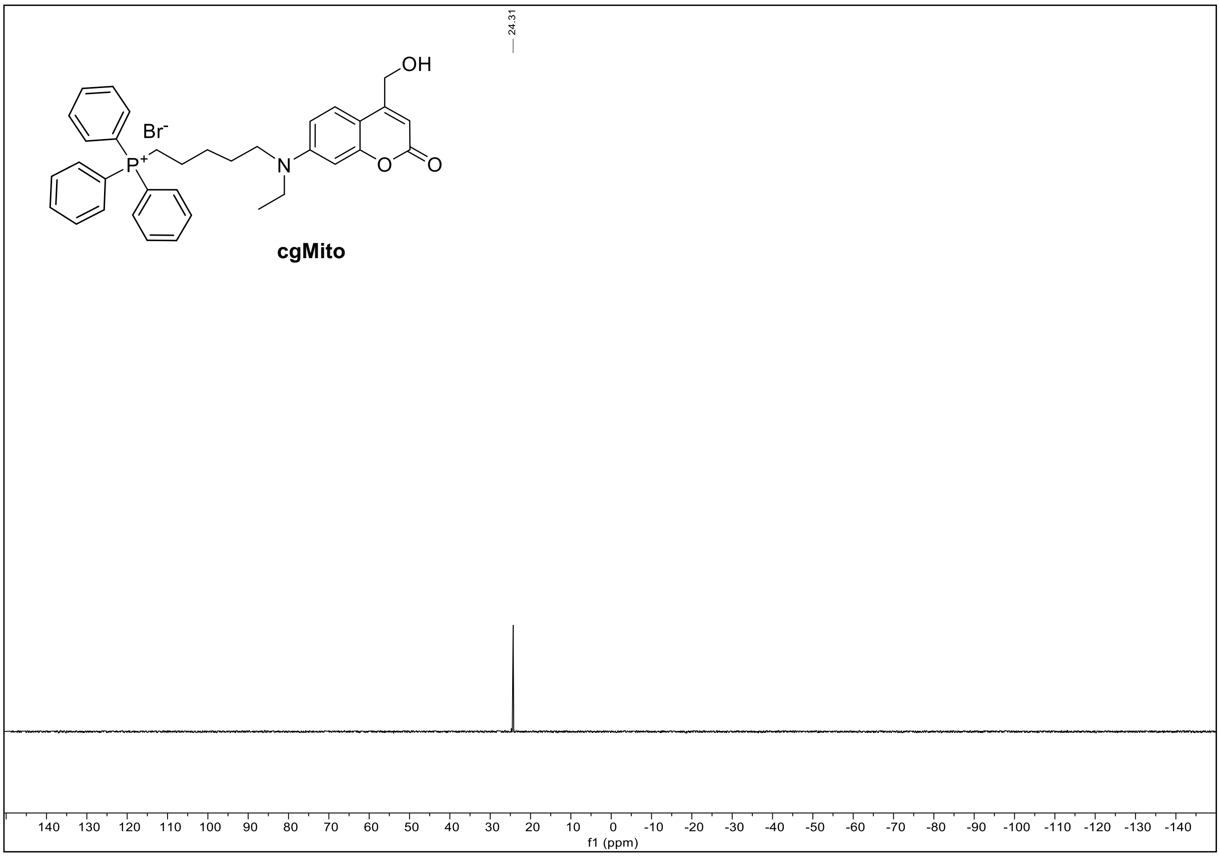

^{31}P-NMR spectrum of **cgMito**

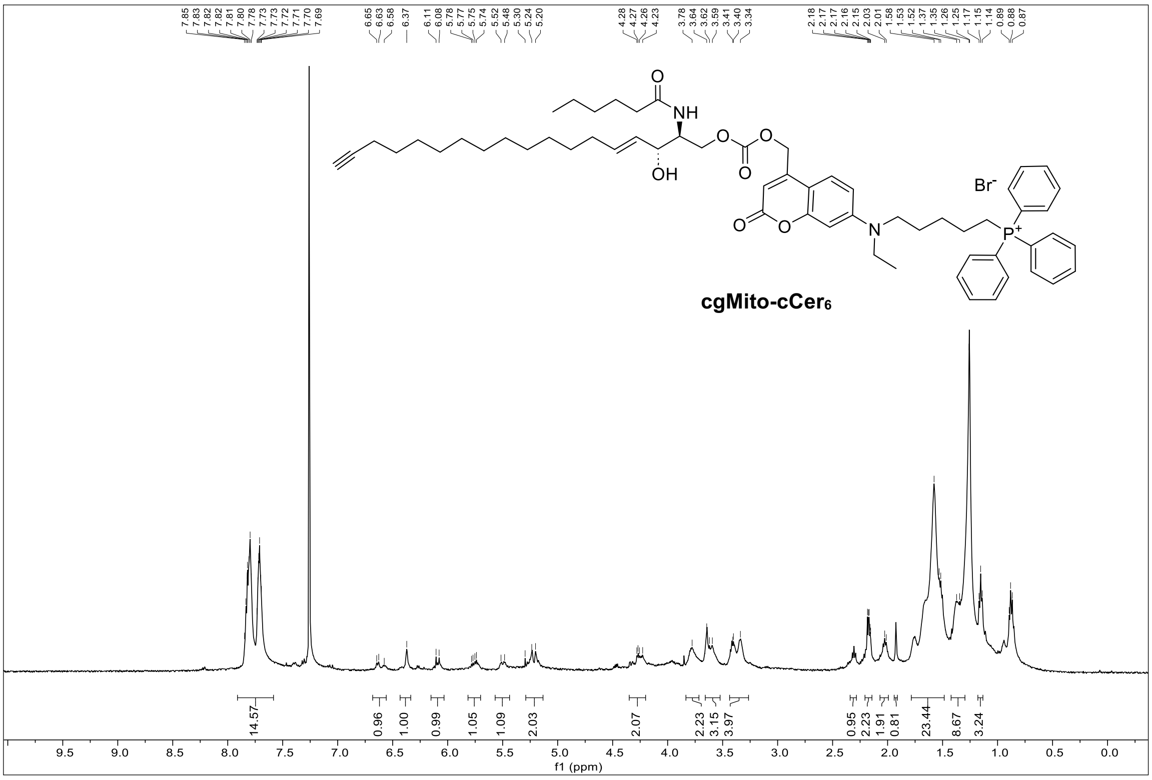

^{1}H-NMR spectrum of **cgMito-cCer$_6$**

1.4 References

Chu, Q.; Makhlouf Brahmi, M.; Solovyev, A.; Ueng, S.-H.; Curran, D. P.; Malacria, M.; Fensterbank, L.; Lacôte, E. Ionic and organometallic reductions with N-heterocyclic carbene boranes. *Chem. Eur. J.*, **2009**, *15*, 12937-12940. doi: 10.1002/chem.200902450.

Deanel K. J.; Summers, R. L.; Lehane, A. M.; Martin, R. E. and Barrow R. A., Chlorpheniramine Analogues Reverse Chloroquine Resistance in Plasmodium falciparum by Inhibiting PfCRT, *ACS Med.Chem. Lett.,* **2014**, 5(5), 576-581.

Fenlon, E. E.; Ito, B. R. The thread & cut method: syntheses of molecular knot precursors. *Eur. J. Org. Chem*., **2008**, *18*, 3065–3068. doi.org/10.1002/ejoc.200800387.

Gan, Y.; Wang, P.; Spencer, T. A. Synthesis of benzophenone-containing fatty acids. *J. Org. Chem.*, **2006**, *71*, 9487–9490. doi.org/10.1021/jo061617n.

Kol, M.; Novak, A. J. E.; Morstein, J., Schröer, C., Sokoya, T.; Mensin, J.; Korneev, S. M.; Trauner, D.; Holthuis, J. C. M. Optical control of sphingolipid biosynthesis using photoswitchable sphingosines. *J. Lipid Res*., **2025**, *66*, 100724.

Lin, Q.; Bao, C.; Fan, G.; Cheng, S.; Liu, H.; Liu, Z.; Zhu, L. 7-Amino coumarin based fluorescent phototriggers coupled with nano/bio-conjugated bonds: synthesis, labeling and photorelease. *J. Mater. Chem*., **2012**, *22*, 6680-6688. doi: 10.1039/c2jm30357d

Mina, J. G.; Mosely, J. A.; Ali, H. Z.; Denny, P. W.; Steel, P. G. Exploring *Leishmania major* inositol phosphorylceramide synthase (*Lmj*IPCS): insights into the ceramide binding domain. *Org. Biomol. Chem*., **2011**, *9*, 1823-1830. doi: 10.1039/c0ob00871k.

Nguyen, T. B.; Castanet, A.-S.; Nguyen, T.-H.; Nguyen, K. P. P.; Bardeau, J.-F.; Gibaud, F.; Mortier, J. Synthesis of model long-chain ω-alkenyltrichlorosilanes and triethoxysilanes for the formation of self-assembled monolayers, *Tetrahedron*, **2006**, *62*, 647–651. doi.org/10.1016/j.tet.2005.10.003.

Ohwada, T.; Kojima, D.; Kiwada, T.; Futaki, S.; Sugiura, Y.; Yamaguchi, K.; Nishi, Y.; Kobayashi, Y. *α,α*-Disubstituted glycines bearing a large hydrocarbon ring: peptide self-assembly through hydrophobic recognition. *Chem. Euro. J.*, **2004**, *10*, 617–626. doi.org/10.1002/chem.200305492.

Predeus, A. V.; Gopalsamuthiram, V.; Staples, R. J.; Wulff, W. D. Rational synthesis for all all-homocalixarenes. *Angew. Chem. Int. Ed*., **2013**, *52*, 911-915. doi: 10.1002/anie.201206785.

Yamamoto, T.; Hasegawa, H.; Hakogi, T.; Katsumura, S. Versatile synthetic method for sphingolipids and functionalized sphingosine derivatives via olefin cross metathesis. *Org.Lett*., **2006**, *8*, 5569-5572. doi: 10.1021/ol062258l

Vuilleumier, J.; Gaulier, G.; De Matos, R.; Ortiz, D.; Menin, L.; Campargue, G.; Mas, C.; Constant, S.; Le Dantec, R.; Mugnier, Y.; Bonacina, L.; Gerber-Lemaire, S. Two-photon-triggered photorelease of caged compounds from multifunctional harmonic nanoparticles. *ACS Appl. Mater. Interfaces*., **2019**, *11*, 27443-27452. doi: 10.1021/acsami.9b07954.
